# Consensus statements from the Hong Kong Urological Association and the Hong Kong Society of Uro-Oncology on the management of muscle-invasive and advanced urothelial carcinoma

**DOI:** 10.3389/fonc.2025.1564487

**Published:** 2025-05-08

**Authors:** Darren M.C. Poon, Peter K.F. Chiu, Marco T.Y. Chan, Brian S.H. Ho, K.S. Law, Angus K.C. Leung, Clarence L.H. Leung, R. Na, Kenneth C.W. Wong, Philip Y. Wu, Philip W.K. Kwong, Jeremy Y.C. Teoh

**Affiliations:** ^1^ Department of Clinical Oncology, State Key Laboratory of Translational Oncology, Sir YK Pao Centre for Cancer, Hong Kong Cancer Institute, The Chinese University of Hong Kong, Hong Kong, Hong Kong SAR, China; ^2^ Comprehensive Oncology Centre, Hong Kong Sanatorium and Hospital, Hong Kong, Hong Kong SAR, China; ^3^ S.H. Ho Urology Centre, Department of Surgery, The Chinese University of Hong Kong, Hong Kong, Hong Kong SAR, China; ^4^ Division of Urology, Department of Surgery, Tuen Mun Hospital, Hong Kong, Hong Kong SAR, China; ^5^ Division of Urology, Department of Surgery, Queen Mary Hospital, Hong Kong, Hong Kong SAR, China; ^6^ Department of Clinical Oncology, Princess Margaret Hospital, Hong Kong, Hong Kong SAR, China; ^7^ AMO Oncology Centre, Hong Kong, Hong Kong SAR, China; ^8^ eFLO Urology, Hong Kong, Hong Kong SAR, China; ^9^ Division of Urology, Department of Surgery, The University of Hong Kong, Hong Kong, Hong Kong SAR, China; ^10^ Department of Clinical Oncology, The Chinese University of Hong Kong, Hong Kong, Hong Kong SAR, China; ^11^ Department of Clinical Oncology, Pamela Youde Nethersole Eastern Hospital, Hong Kong, Hong Kong SAR, China; ^12^ Hong Kong Integrated Oncology Centre, Hong Kong, Hong Kong SAR, China

**Keywords:** antibody-drug conjugate, carboplatin, chemotherapy, cisplatin, en bloc resection, immune checkpoint inhibitor, nephron-sparing surgery, oligometastasis

## Abstract

**Background:**

Muscle-invasive and advanced urothelial carcinoma (UC) are notorious for their high propensity for recurrence and metastasis. Recent advances in novel medications, surgical procedures, and radiotherapy techniques have substantially transformed the treatment landscape of muscle-invasive and advanced UC. It is crucial to navigate the optimal management approaches for muscle-invasive and advanced UC through the increasingly complex matrix of variables.

**Methods:**

Two professional organisations convened a consensus panel of six urologists and six clinical oncologists with extensive experience in treating urological malignancies. They reviewed the literature on the management of i) non-metastatic, muscle-invasive, and locally advanced UC of the bladder; ii) locally advanced upper tract UC (UTUC); and iii) unresectable locally advanced or metastatic UC (mUC). The panel held multiple meetings to discuss and draft consensus statements using the modified Delphi method. Each drafted statement was anonymously voted on by every panellist. A consensus statement was accepted if ≥ 80% of the panellists chose ‘accept completely’ or ‘accept with some reservation’ from the five options, which also included ‘accept with major reservation’, ‘reject with reservation’, and ‘reject completely’.

**Results:**

The panel reached a consensus on 63 statements based on current evidence and expert insights. These statements addressed the considerations for different treatment modalities, including surgical approaches, radiotherapy, radiosensitisers, platinum-based chemotherapy, immune checkpoint inhibitors, and antibody–drug conjugates, in the management of different disease entities, including muscle-invasive UC of the bladder, cN1 disease, locally advanced UTUC, unresectable locally advanced/mUC, and oligometastatic bladder cancer.

**Conclusion:**

These consensus statements are anticipated to serve as a practical recommendation for clinicians in Hong Kong, and possibly the Asia-Pacific region, regarding the management of muscle-invasive and advanced UC.

## 1 Introduction

Bladder cancer (BC) is among the 10 most common malignancies worldwide (1). In Hong Kong, the annual incidence of BC is ~400 patients, most of whom are men aged 55–70 years (2). Urothelial carcinoma (UC) is the typical histological subtype of BC (3). By tumour invasiveness, 75% and 25% of BCs are classified into non–muscle-invasive BC (NMIBC) and muscle-invasive BC (MIBC), respectively; the latter is associated with a far higher risk of metastasis than the former (3). Although mostly arising in the bladder, UC can also develop in the upper urinary tract, which includes the renal pelvis and ureter; upper tract UC (UTUC) accounts for 5–10% of all UC cases and locally advanced disease is frequently found (60%) at the time of radical surgery (4). Recent advances in novel medications (e.g. antibody–drug conjugates and immunotherapy [IO] with immune checkpoint inhibitors [ICIs]), surgical procedures, and radiotherapy (RT) techniques have greatly transformed the treatment landscape of MIBC, locally advanced UTUC, and inoperable/metastatic UC (mUC). In order to enhance patient care, it is important to navigate the optimal management approaches for muscle-invasive and advanced UC through the increasingly complicated matrix of variables.

## 2 Methods

Between May and September 2024, the Hong Kong Urological Association and the Hong Kong Society of Uro-Oncology convened a panel of experts to discuss current evidence and their insights regarding contemporary therapies for muscle-invasive and advanced UC in a series of meetings, with the ultimate goal of establishing consensus statements using the modified Delphi method (5). The panel included six urologists and six clinical oncologists with > 10 years of experience treating patients with urological malignancies in public or private institutions. The panel discussions focused on the management of locally advanced/MIBC, locally advanced UTUC, and unresectable locally advanced/mUC (**Table 1**). The panel commissioned a medical writing agency to search the PubMed database for publications that were pertinent to the discussion areas. In order to address the three areas of focus, the panel was divided into three groups (**Table 1**), each of which presented the relevant literature and any additional appropriate data, and shared their clinical experiences.

**Table 1:** Discussion areas.

Main part	Subpart	Number of consensus statements accepted	Number/specialty of responsible panellists
1. Management of non-metastatic muscle-invasive and locally advanced UC of the bladder	1.1. Candidacy for RC, RT, or TMT	16	4 urologists + 2 clinical oncologists
	1.2. Role of neoadjuvant and adjuvant systemic pharmacotherapy	7	
	1.3. Role of RT in muscle-invasive BC	3	
	1.4. Surgical aspects of TMT	3	
	1.5. Management of cN1 disease	3	
	1.6. Follow-up and monitoring	2	
2. Management of locally advanced UTUC	2.1. Considerations for prescribing neoadjuvant or adjuvant platinum-based chemotherapy or immunotherapy in patients with UTUC	11	1 urologist + 1 clinical oncologist
	2.2. Optimal follow-up schedule in patients with UTUC	3	
3. Management of unresectable locally advanced or metastatic UC	3.1. Initial treatment choice	5	1 urologist + 3 clinical oncologists
	3.2. Subsequent treatment approach	6	
	3.3. Management of oligometastatic BC	4	

BC, bladder cancer; RC, radical cystectomy; RT, radiotherapy; TMT, trimodal bladder-sparing therapy; UC, urothelial carcinoma; UTUC, upper tract urothelial carcinoma.

Based on the discussions, each group drafted consensus statements for their respective area. At the last meeting, the panel reviewed, modified, and finalized the consensus statements. Each statement was voted on anonymously by every panellist based on the practicability of the recommendation. A consensus statement was accepted only if ≥ 80% of the panel chose ‘accept completely’ or ‘accept with some reservation’ from the five options, which also included ‘accept with major reservation’, ‘reject with reservation’, and ‘reject completely’. The agreement threshold of 80% followed the common practice of international Delphi consensus studies (6, 7). Appendix S1 details full voting records for all accepted and rejected statements.

## 3 Results

A total of 63 consensus statements were accepted and are summarised in **Table 1**. The rationale for each is described below.

### 3.1 Part 1 – Management of non-metastatic muscle-invasive and locally advanced UC of the bladder

**Figure 1:**
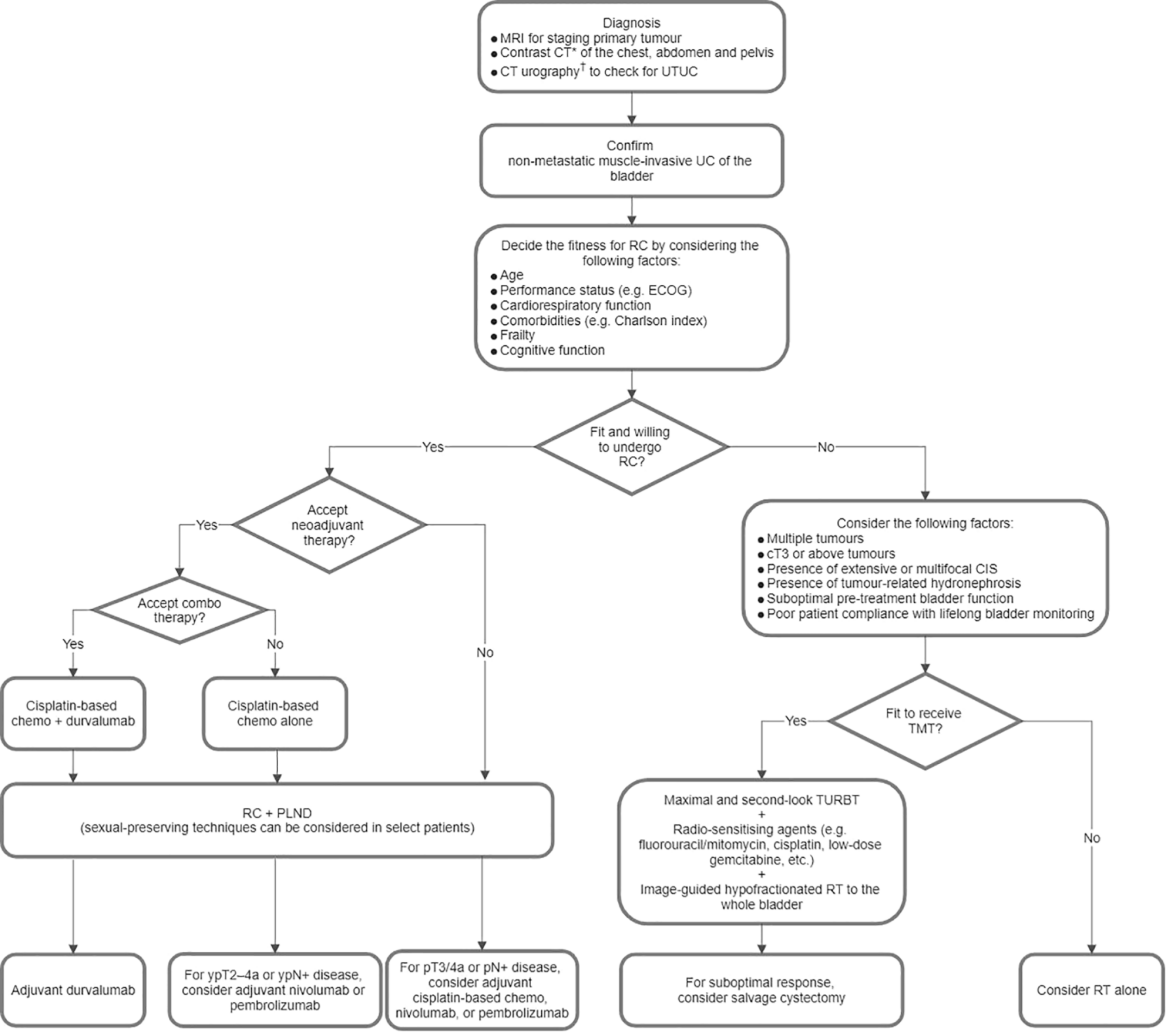
Proposed treatment algorithm for non-metastatic muscle-invasive urothelial carcinoma (UC) of the bladder. *Positron emission tomography-computed tomography (CT) or contrast magnetic resonance imaging (MRI) of the abdomen and pelvis is an alternative for screening nodal and distant metastases. ^†^Magnetic resonance urography is an alternative. Chemo, chemotherapy; CIS, carcinoma *in situ*; ECOG, Eastern Cooperative Oncology Group; PLND, pelvic lymph node dissection; RC, radical cystectomy; RT, radiotherapy; TMT, trimodal bladder-sparing therapy; TURBT, transurethral resection of bladder tumour; UTUC, upper tract urothelial carcinoma.

**Figure 1** illustrates a proposed treatment algorithm for MIBC that was derived from the consensus statements from Parts 1.1 to 1.4.

#### 3.1.1 Part 1.1 Candidacy for radical cystectomy, RT, and trimodal bladder-sparing therapy

Statement 1: Radical cystectomy (RC) with standard pelvic lymph node dissection (PLND) is the standard treatment for patients with non-metastatic MIBC.

In a randomised phase III trial (8), patients with locally resectable T1G3 or muscle-invasive UC (T2–T4aM0) were randomised to receive limited PLND (obturator, and internal and external iliac nodes; n = 203) or extended PLND (additionally deep obturator, common iliac, presacral, paracaval, interaortocaval, and para-aortal nodes up to the inferior mesenteric artery; n = 198) at the time of RC. Both groups had similar rates of recurrence-free survival (RFS), cancer-specific survival (CSS), and overall survival (OS). The extended PLND group had a higher rate of Clavien grade ≥ 3 lymphoceles (8.6% vs. 3.4% in the limited PLND group; P = 0.04) (8). Notably, this negative study result may be attributable to the high median numbers of lymph nodes (LNs) dissected in both groups (19 in the limited arm; 31 in the extended arm) and the inclusion of patients with T1G3 tumours (8). Another randomised phase III study, SWOG S1011, demonstrated that, with a median follow-up of 6.1 years, compared with extended PLND (up to at least the aortic bifurcation and including common iliac, pre-sciatic, and pre-sacral nodes; n = 292), standard PLND (obturator, and internal and external iliac nodes; n = 300) yielded similar rates of disease-free survival (DFS) and OS, with a shorter median operative time (5.3 vs. 5.9 hours), less median blood loss (600 vs. 700 mL), a lower rate of deep venous thrombosis (6.1 vs. 9.8%), and a lower rate of Grade 3/4 adverse events (8 vs. 16%), in patients with MIBC (T2, 71%; T3–4a, 29%) (9). These two studies suggest that extended PLND does not offer clinical benefits over standard PLND during RC.

Statement 2: For male patients, cystectomy with sexual-preserving techniques should only be considered in patients with organ-confined disease and in those without tumours at the prostate, prostatic urethra, or bladder neck.

Statement 3: For female patients, sexual organ-preserving cystectomy should only be considered in patients with organ-confined disease.

Statement 4: Patients who want to preserve sexual function should be counselled on various sexual-preserving surgical techniques.

For male patients with MIBC, organ-sparing cystectomy is mainly aimed at preserving sexual function. However, for female patients with MIBC, organ-sparing cystectomy can also help preserve pre-menopausal hormonal homeostasis, reduce the risk of urinary retention by supporting the construction of the neobladder, and avoid post-operative prolapse.

Organ-sparing methods for male patients include prostate-sparing cystectomy, capsule-sparing cystectomy, seminal-sparing cystectomy, and nerve-sparing cystectomy. A systematic review analysed the oncological and functional outcomes of sexual function-preserving cystectomy compared with standard RC in men with MIBC (10). Notably, the included studies were heterogeneous, with no clear comparisons among different preservation techniques. According to the European Association of Urology (EAU) guidelines (11), prostate-sparing and capsule-sparing cystectomy may be offered to highly selected men with negative prostatic urethral and transrectal prostate biopsies, who may wish to preserve fertility or sexual function. Furthermore, nerve-sparing and prostate-sparing procedures may offer similar rates of sexual function preservation in men with MIBC. Patients who express a desire to preserve sexual function should be carefully counselled.

Data on pelvic organ-preserving cystectomy for female patients remain immature. A systematic review (12) revealed that well-selected female patients with MIBC who underwent pelvic organ-preserving cystectomy with orthotopic neobladder had a median sexual satisfaction score of 88.5/100, a CSS of 70–100% in 3–5 years, and an OS of 65–100% in 3–5 years, with acceptable rates of continence (daytime, 58–100%; nighttime, 42–100%) and self-catheterisation (9.5–78%). Although the organ-preserving approach appears to offer similar oncological outcomes and better sexual/urinary function compared with standard RC in female patients, poor reporting and large heterogeneity between studies in the systematic review should be considered. The EAU has recommended that, among female patients with MIBC, only those with organ-confined disease are eligible for organ-preserving cystectomy (11). Both male and female patients should be carefully counselled before deciding to undergo organ-preserving cystectomy.

Statement 5: A positive prostatic urethral biopsy in transurethral resection (TUR) of bladder tumour (TURBT) does not correlate with the final margin and should not exclude patients from consideration for orthotopic bladder reconstruction.

A study at the McGill University Health Center included 252 men who underwent RC with ileal neobladder, with 245 having data on pre-operative TUR prostatic urethral biopsy and/or intra-operative frozen section of the urethra (13). With respect to final margins, TUR prostatic urethral biopsy yielded a positive predictive value of 12.5% and a negative predictive value of 99.4% (13). Frozen section had a negative predictive value of 100% regarding final margins (13). There was a weak correlation (68%) between TUR biopsy findings and frozen section margins (13). In summary, a negative TUR biopsy or frozen section can identify patients who should avoid urethrectomy, but a positive TUR biopsy cannot confirm the need for urethrectomy and thus should not serve as a basis to preclude orthotopic bladder reconstruction.

Statement 6: Before undergoing RC, the Enhanced Recovery After Surgery (ERAS) approach should be considered to reduce the risks of post-operative ileus and venous thromboembolism.

The ERAS protocol is aimed at maintaining pre-operative organ function and facilitating early recovery after surgery (14). The ERAS approach for RC ± neobladder includes pre-operative carbohydrate loading and reduced bowel preparation, intra-operative use of short-acting anaesthetic agents, and comprehensive post-operative measures, such as early mobilisation and optimal pain management (14). A retrospective study of patients who underwent open RC showed that those on an ERAS protocol (n = 124) experienced a significantly lower incidence of post-operative ileus (7.3% vs. 22.2%; P = 0.003) compared with those on a traditional recovery protocol (n = 81) (15). In a pre- and post-intervention analysis of 319 patients who underwent RC, the introduction of an ERAS measure – a perioperative venous thromboembolism (VTE) prophylaxis programme (including administration of enoxaparin post-discharge for 28 days) – significantly reduced the rate of 30-day post-operative VTE (0.9% vs. 6.2%; P = 0.04) compared with the absence of the programme (16).

Statement 7: To decide whether a patient is fit for RC, the following factors should be considered:

a. Age;
b. Performance status [e.g. Eastern Cooperative Oncology Group (ECOG)];
c. Cardiorespiratory function;
d. Comorbidities (e.g. Charlson index);
e. Frailty; and
f. Cognitive function.

In general, younger patients are anticipated to have better survival outcomes and quality of life post-surgery (17, 18). In addition to age, related factors, such as performance status, organ function, comorbidities, and frailty, are independently associated with the incidences of complications, disease progression, cancer-specific mortality, and overall mortality post-surgery (17, 19, 20). BC is predominantly a geriatric disease; however, age should not be the only determinant of whether to undergo RC. Instead, the treatment decision should be based upon a holistic assessment of the listed factors, as well as sufficient pre-operative discussions with patients and carers alike.

Statement 8: RT alone should only be a treatment option for patients who are unfit for both RC and concurrent chemo-irradiation.

Pooling data from three randomised trials, a systematic review reported that the mean OS rates at 3 and 5 years were 45% and 36% for RC, and 28% and 20% for RT alone, in patients with MIBC (21). In an analysis of the U.S. National Cancer Data Base, concurrent chemo-RT (n = 630) was significantly associated with a higher 2-year OS rate (56% vs. 42%; P < 0.0001) compared with RT alone (60–70 Gy; n = 739) in patients with MIBC aged ≥ 80 years (22). With inferior oncological outcomes compared with RC or concurrent chemo-irradiation, RT alone is not a standard of care for MIBC and should only be considered when neither surgery nor concurrent chemotherapy is applicable.

Statement 9: TMT should include all three modalities, i.e. maximal TURBT, radiosensitising agents (e.g. chemotherapy), and RT.

A study at the Massachusetts General Hospital included 348 patients who underwent concurrent chemo-RT after maximal TURBT for cT2–4a disease, with a median follow-up of 7.7 years for surviving patients (23). Visibly complete TURBT was significantly associated with better clinical outcomes in terms of the rates of complete response (CR) (79% vs. 57%; P < 0.001), OS (5-year, 57% vs. 43%; 10-year, 39% vs. 29%; P = 0.003), DFS (5-year, 68% vs. 56%; 10-year, 63% vs. 51%; P = 0.03), and salvage cystectomy (22% vs. 42%; P < 0.001) compared with visibly incomplete TURBT (23). These data highlight the importance of maximal TURBT in optimising the outcomes of TMT.

TMT with maximal TURBT, RT, and radiosensitisers (e.g. cisplatin plus paclitaxel or 5-fluorouracil [5-FU]; mitomycin-C [MMC] plus 5-FU; or gemcitabine) can achieve a median OS rate of up to 75% in 3–5 years among patients maintaining a functional bladder (24). There is no definitive contemporary evidence to support neoadjuvant or adjuvant chemotherapy combined with chemo-RT in TMT (24).

Statement 10: TMT is NOT preferred in patients with the following characteristics:

a. Multiple tumours;
b. cT3 or above tumours;
c. Presence of extensive or multifocal carcinoma *in situ* (CIS);
d. Presence of tumour-related hydronephrosis;
e. Suboptimal pre-treatment bladder function; or
f. Poor patient compliance with lifelong bladder monitoring.

Several studies have provided insights into the patient selection for TMT. In a multicentre retrospective study of patients with muscle-invasive UC of the bladder (25), analyses using propensity score matching (PSM) and inverse probability treatment weighting (IPTW) showed that RC and TMT yielded similar oncological outcomes in terms of 5-year metastasis-free survival (RC, 74% vs. TMT, 74%; P = 0.64 [PSM]; 74% vs. 75%; P = 0.40 [IPTW]) and 5-year CSS (RC, 83% vs. TMT, 85%; P = 0.057 [PSM]; 81% vs. 84%; P = 0.071 [IPTW]). Regarding patient characteristics, 90% of the study participants had cT2 disease, and all had solitary tumours < 7 cm with no or unilateral hydronephrosis, and no extensive or multifocal CIS (25). Two randomised phase III trials, namely BC2001 (26) and BCON (27), demonstrated the RFS benefits of concurrent RT and radiosensitisation (using chemotherapy in BC2001 and hypoxia-modifying therapy in BCON) over RT alone for the treatment of MIBC. The participants in the intervention arms in both studies had similar characteristics, including ~80% of patients having pT1–2 disease and ~40% having incomplete resection (26, 27). This relatively high proportion of patients with incomplete resection suggests that such a factor does not necessarily hinder the use of TMT. Additionally, a retrospective analysis revealed that the extent of TURBT did not independently affect OS, CSS, metastasis-free survival, or DFS in patients treated with chemo-RT for MIBC (n = 757; 66% complete TURBT vs. 34% incomplete TURBT) at 5 years (28). Indeed, the panellists did not reach a consensus on a draft statement that TMT is not preferred in patients with incomplete resection. To enhance the success of TMT, patients are preferred to have optimal pre-treatment bladder function (29) and good adherence to long-term follow-up to address the persistent potential for recurrence of the BC (11).

Statement 11: Magnetic resonance imaging (MRI) is the preferred imaging technique for staging the primary tumour.

A historical study demonstrated that MRI (T1/2-weighted or gadolinium-enhanced) appeared to be more accurate (75% vs. 55%) than contrast computed tomography (CT) for staging patients with histologically proved UC (n = 36) (30). Recently, the 5-point Vesical Imaging-Reporting and Data System (VI-RADS) score was introduced to provide standard protocols and reporting criteria (including size, location, multiplicity, and morphology) in the assessment of the presence of muscle invasion when staging UC using multiparametric MRI (31). A VI-RADS score of 5 can differentiate extravesical disease from muscle-confined UC before TURBT, with 90.2% sensitivity, 98.1% specificity, a positive predictive value of 94.9%, and a negative predictive value of 96.4% for the detection of extravesical disease (32).

Statement 12: Contrast CT of the chest, abdomen and pelvis should be considered for the screening of nodal and distant metastases.

Contrast CT is the imaging tool of choice for the detection and characterisation of pulmonary nodules (33). For the detection of nodal metastases, CT and MRI show similar results, with the sensitivity ranging from 48% to 87% (11). Barentsz et al. suggested that pelvic nodes and abdominal nodes with minimal axial diameters of > 8 mm and > 10 mm, respectively, on CT or MRI should be regarded as pathologically enlarged (34).

Statement 13: CT urography should be considered to evaluate the presence of any UTUC.

In a study evaluating the diagnostic accuracy of CT urography in a selected series of 106 patients with haematuria, it was shown that CT urography provided a sensitivity of 97%, a specificity of 93%, a positive predictive value of 79%, and a negative predictive value of 99% for the detection of UTUC (35). The sensitivity of CT urography appears to be markedly higher than that of magnetic resonance (MR) urography (62.9%–74.3%) (36). Therefore, CT urography is the preferred imaging tool to screen for UTUC.

Statement 14: Positron emission tomography (PET)-CT can be considered for the screening of nodal and distant metastases.

In a systematic review and meta-analysis (37), the pooled data from 785 patients with newly diagnosed UC across 14 studies showed that ^18^F-fluorodeoxyglucose (FDG) PET-CT provided a sensitivity of 57% and a specificity of 92% for the detection of nodal metastases. As such, PET-CT performs similarly to CT or MRI in screening for nodal disease. Despite a lack of data on the performance of FDG PET-CT in staging distant metastases, it appears to be comparable to CT and MRI for the accurate detection of pulmonary and hepatic metastases (38).

Statement 15: Contrast MRI of the abdomen and pelvis is a suitable alternative for the screening of LN and visceral metastases, and MR urography for the detection of UTUC.

As mentioned under Statement 12, MRI and CT yield similar results for the detection of nodal metastases. Notably, while MRI has a limited role in the detection of pulmonary metastases, it appears to offer similar sensitivity to contrast CT for the diagnosis of hepatic metastases (38). Regarding the detection of UTUC, MR urography should only be used in patients who are contraindicated for CT urography, primarily due to radiation or iodinated contrast agents (11).

Statement 16: Routine screening for asymptomatic brain or bone metastases is not recommended.

Because brain and bone metastases are rare in patients with UC, only those who are obviously symptomatic should undergo relevant imaging (11).

#### 3.1.2 Part 1.2 Role of neoadjuvant and adjuvant systemic pharmacotherapy

Statement 1: In patients with cT2–4a N0M0 disease who will undergo RC and are cisplatin-eligible, the option of neoadjuvant cisplatin-based chemotherapy plus perioperative durvalumab IO should be offered.

Findings from the recent randomised phase III NIAGARA trial (39) suggested that neoadjuvant durvalumab plus gemcitabine–cisplatin (GemCis) chemotherapy followed by RC and adjuvant durvalumab should be the new standard of care for the management of MIBC. This study included 1,063 cisplatin-eligible patients with MIBC, with 60% having > T2N0 disease and 73% having high programmed death-ligand 1 (PD-L1) expression (39). At 24 months, compared with neoadjuvant GemCis followed by RC, the investigational regimen significantly improved the estimated rate of event-free survival at 24 months (EFS, 67.8% vs. 59.8%; hazard ratio [HR], 0.68; 95% confidence interval [CI], 0.56–0.82; P < 0.001) and the estimated 24-month OS (82.2% vs. 75.2%; HR, 0.75; 95% CI, 0.59–0.93; P = 0.01) in the overall population (39). Neoadjuvant durvalumab did not delay RC or affect the feasibility of undergoing or completing RC (39).

Statement 2: There is currently a lack of level I evidence to support the use of IO, without chemotherapy, in the neoadjuvant setting.

The use of neoadjuvant IO for MIBC has only been investigated in phase II single-arm trials. The PURE-01 study showed that, among 50 patients with cT ≤ 3bN0 MIBC who received neoadjuvant pembrolizumab followed by RC, 21 (42%) achieved a pathological CR (40). Due to the protocol updates in sample size and statistical assumptions, the final study cohort of PURE-01 included 155 patients (41). The 3-year updated results showed that 74.4% and 83.8% of the overall population (92.3% underwent RC) attained EFS and OS, respectively, with PD-L1 expression being the strongest predictor of sustained response post-RC (41).

Another trial on neoadjuvant ICI therapy for MIBC was ABACUS. It demonstrated that, among 95 patients who received neoadjuvant atezolizumab (followed by RC in 91.6%), 31% achieved a pathological CR (42). The 2-year final results showed that 68% and 77% of the overall population achieved DFS and OS, respectively; however, subgroup analyses did not identify useful biomarkers (43).

Statement 3: Adjuvant chemotherapy should only be considered in patients who have not received neoadjuvant chemotherapy and have advanced disease, i.e. pT3/4 and/or pN+ disease.

The role of adjuvant chemotherapy in MIBC has been debated, especially since the release of the NIAGARA trial results (39). Previously, a systematic review and meta-analysis supported the use of adjuvant chemotherapy in selected patients (44). Based on the pooled data from nine randomised controlled trials that included 945 patients, most of whom had T3/4 disease, adjuvant cisplatin-based chemotherapy after RC significantly improved OS (HR, 0.77; 95% CI, 0.59–0.99; P = 0.049) and DFS (HR, 0.66; 95% CI, 0.45–0.91; P = 0.014) compared with RC alone, and the DFS benefit was more marked in patients with nodal disease (P = 0.010) (44). However, these results should be interpreted cautiously because three of the included studies showed that adjuvant chemotherapy (with cisplatin-based combinations, single-agent cisplatin, and GemCis) did not significantly improve DFS compared with RC alone (44). The inclusion of early-terminated studies and a lack of data analysis at the individual patient level are other caveats of the review (45).

Statement 4: Adjuvant nivolumab therapy can be considered in patients with ypT2–4a or ypN+ MIBC after neoadjuvant cisplatin-based chemotherapy (without IO).

Statement 5: Adjuvant pembrolizumab therapy can be considered in patients with ypT2–4a or ypN+ MIBC after neoadjuvant cisplatin-based chemotherapy (without IO).

Statement 6: Adjuvant nivolumab therapy can be considered in patients with pT3–4a or pN+ MIBC who have not received neoadjuvant cisplatin-based chemotherapy.

Statement 7: Adjuvant pembrolizumab therapy can be considered in patients with pT3–4a or pN+ MIBC who have not received neoadjuvant cisplatin-based chemotherapy.

The randomised placebo-controlled phase III CheckMate 274 trial investigated the efficacy and safety of adjuvant nivolumab post-RC in patients with ypT2–4a or ypN+ MIBC who received neoadjuvant cisplatin-based chemotherapy, and patients with pT3–4a or pN+ MIBC who did not receive neoadjuvant cisplatin-based chemotherapy and refused or were ineligible for adjuvant cisplatin-based chemotherapy (46, 47). Consistent with the initial results (46), it was shown, after extended follow-up (47), that adjuvant nivolumab continued to improve the median DFS in the intention-to-treat (ITT) population (22.0 vs. 10.9 months; HR, 0.71; 95% CI, 0.58–0.86) and the subgroup with PD-L1 ≥ 1% (52.6 vs. 8.4 months; HR, 0.52; 95% CI, 0.37–0.72) compared with placebo. Adjuvant nivolumab was also associated with better interim OS in the ITT population (median, 69.5 vs. 50.1 months; HR, 0.76; 95% CI, 0.61–0.96) and the PD-L1–positive subgroup (median, both not reached; HR, 0.56; 95% CI, 0.36–0.86) compared with placebo (47). Prespecified subgroup analyses of the ITT population showed that the OS benefits of adjuvant nivolumab over placebo were more apparent in patients with N+ disease (HR, 0.69; 95% CI, 0.51–0.93), patients with pT3 disease (HR, 0.67; 95% CI, 0.49–0.91), and patients who received neoadjuvant cisplatin (HR, 0.71; 95% CI, 0.51–0.99) (47). The panel generally agreed that adjuvant nivolumab can be used in all eligible patients, regardless of PD-L1 status, for two reasons derived from CheckMate 274: 1) DFS among the ITT population was a primary endpoint, and it was met; 2) in subgroup analyses, the DFS benefit of adjuvant nivolumab over placebo was shown in patients with both PD-L1 ≥ 1% and < 1%, with no significant difference reported.

With a similar design to CheckMate 274, the AMBASSADOR trial investigated the effects of adjuvant pembrolizumab in 702 post-RC patients (~60% received neoadjuvant therapy and ~60% were PD-L1–positive) (48). At 42 months, adjuvant pembrolizumab significantly improved median DFS (29.6 vs. 14.2 months; HR, 0.73; 95% CI, 0.59–0.90; P = 0.003) compared with observation (48). The benefits of adjuvant pembrolizumab were consistent in PD-L1–positive (HR, 0.81) and PD-L1–negative (HR, 0.71) subgroups (48). For OS, the interim analysis at 3 years showed that there was no significant difference between the two arms (60.8 months for pembrolizumab vs. 61.9 months for observation; HR, 0.98; 95% CI, 0.76–1.26), possibly because a high proportion (52.2%) of patients in the observation arm received ICI therapy after disease recurrence (48).

#### 3.1.3 Part 1.3 Role of RT in MIBC

Statement 1: When TMT is considered, image-guided hypofractionated RT to the whole bladder concurrent with radiosensitising agents should be the standard of care.

To compare outcomes of two commonly used RT schedules for MIBC, i.e. 64 Gy in 32 fractions over 6.5 weeks and a hypofractionated schedule of 55 Gy in 20 fractions over 4 weeks, Choudhury et al. conducted a meta-analysis of individual patient data from two randomised phase III trials in which participants received RT alone versus RT with chemotherapy (in the BC2001 trial) or RT with hypoxia-modifying therapy (in the BCON trial) for the management of locally advanced UC (T1G3 or T2–T4 N0M0) (49). The analysis included 782 patients with known RT schedules (48% received 64 Gy in 32 fractions; 52% received 55 Gy in 20 fractions) (49). With a median follow-up of 120 months, the hypofractionated schedule was associated with a lower risk of invasive locoregional recurrence (adjusted HR, 0.71; 95% CI, 0.52–0.96) and had a similar toxicity profile (adjusted risk difference, –3.37%; 95% CI, –11.85 to 5.10) compared with the standard schedule (49). The hypofractionated schedule should be adopted as the standard of care for TMT in patients with MIBC.

For RT to UC, the image-guided approach is recommended to enhance tumour control and reduce toxicity by addressing the inter/intra-fractional internal organ motion due to changes in bladder volume with bladder wall deformations (50). A ‘plan of the day’ adaptive RT technique using cone-beam CT and/or MRI can increase the accuracy of bladder irradiation by accounting for individual intra-pelvic anatomical variations (50). There is currently no strong evidence to support partial bladder irradiation; therefore, whole-bladder irradiation remains the standard of care.

Statement 2: Radiosensitising agents (e.g. fluorouracil/mitomycin, cisplatin, low-dose gemcitabine, etc.) can be concurrently given with RT in TMT.

As discussed in Part 1.1, Statement 9, a radiosensitising agent is an essential component of TMT for MIBC. According to the National Comprehensive Cancer Network (NCCN) in the USA (51), several regimens can serve as radiosensitisers, including cisplatin alone, low-dose gemcitabine, 5-FU plus MMC, cisplatin plus paclitaxel, and cisplatin plus 5-FU. One panellist shared that low-dose weekly gemcitabine may be a more tolerable regimen, especially in patients with suboptimal renal function or borderline performance status.

Statement 3: There is currently a lack of evidence to support the use of adjuvant RT after RC.

A multicentre phase II trial investigated the toxicity and efficacy of adjuvant RT after RC in 72 patients with high-risk MIBC, i.e. ≥ pT3 disease ± lymphovascular invasion, < 10 LNs removed, pathologically positive LNs, or positive surgical margins (52). The adjuvant RT was delivered with an intensity-modulated approach to the pelvic LNs ± cystectomy bed, with a dose of 50 Gy in 25 fractions (52). With a median follow-up of 18 months, 42 (61%) patients developed acute grade 2 gastrointestinal toxicity, and 53% (9/17) of patients with a neobladder experienced acute grade 2 urinary toxicity (52). The 2-year rates of local relapse-free survival, OS, and bladder CSS were 83%, 52%, and 62%, respectively (52).

Currently, the evidence for adjuvant RT remains limited. The role of adjuvant RT in MIBC will be further examined in the ongoing phase III BART trial, in which patients with high-risk MIBC (≥ pT3, pN+, positive margins and/or nodal yield < 10, or neoadjuvant chemotherapy given for cT3/T4/N+ disease) are being randomised to observation or adjuvant RT, with the primary endpoint being 2-year locoregional RFS (53). The adjuvant RT will be delivered to the cystectomy bed and pelvic nodes using an intensity-modulated approach, to a dose of 50.4 Gy in 28 fractions (53). Notably, as the standard of care for MIBC is shifting to neoadjuvant GemCis/durvalumab followed by RC and adjuvant durvalumab, it is worth reassessing the role of adjuvant RT in this emerging context.

#### 3.1.4 Part 1.4 Surgical aspects of TMT

Statement 1: If there is a suboptimal response to TMT, salvage cystectomy should be considered.

As an integral part of TMT (54), salvage cystectomy appears to offer promising effectiveness, safety, and tolerability, consistent with primary RC (55, 56). Studies showed that 10–15% of patients who received TMT for MIBC experienced local recurrences within the first 5 years post-TMT, and that timely salvage cystectomy did not compromise oncologic or survival outcomes among these patients (57, 58).

Statement 2: Modified *en bloc* resection may be feasible in TMT for MIBC, depending on MRI findings.

The randomised phase III EB-StaR trial (59) showed that, in patients with NMIBC of ≤ 3 cm, transurethral *en bloc* resection significantly reduced the 1-year recurrence rate compared with standard resection (28.5% vs. 38.1%; P = 0.007). Subsequently, an ongoing phase II single-arm study has begun investigating the feasibility of modified *en bloc* resection for the treatment of bladder tumours > 3 cm in patients with NMIBC and MIBC (60). The modified *en bloc* resection is a hybrid technique that involves piecemeal resection of the exophytic part of the bladder tumour, followed by *en bloc* resection of the tumour base. The composite primary outcome is complete resection for NMIBC and proper staging for MIBC. All patients undergo MRI scanning before modified *en bloc* resection. The NMIBC group are offered second-look TURBT, whereas the MIBC group (with no distant metastases) undergo RC or second-look TURBT if RC is not applicable. All patients have a second MRI before the second surgery. The results of the modified *en bloc* resection specimens are compared with the final pathology results of the second surgery. The study results will further inform the feasibility of modified *en bloc* resection as part of TMT for MIBC based on MRI findings.

Statement 3: Second-look TURBT is recommended in TMT.

A retrospective review by Suer et al. was the first to demonstrate the clinical significance of a second TURBT in patients who plan to undergo TMT for MIBC (61). In the study, a second TURBT identified residual tumours in 29/43 (67.4%) patients and significantly improved the 5-year disease-specific survival rate (68% vs. 41%; P = 0.046) compared with the absence of the procedure (N = 47). Furthermore, the EAU and the European Society of Medical Oncology (ESMO) have recommended considering the likelihood of optimal debulking surgery when assessing patient eligibility for bladder preservation (11). Therefore, it is important to perform a second TURBT to confirm the completeness of resection and, thus, the eligibility for TMT.

#### 3.1.5 Part 1.5 Management of cN1 disease

Statement 1: Neoadjuvant chemotherapy plus perioperative durvalumab IO + RC with PLND (i.e. to the level of the ureteric crossing) should be considered in patients with cN1 disease.

Most panellists considered that the treatment approach for ≥ cN2 disease is generally equivalent to that for mUC (see Part 3), in which local treatment is ineffective. However, similar to MIBC, cN1 disease can still be treated with multimodal therapy; therefore, the panel focused this subsection on the management of cN1 disease.

Previous studies showed that a combination of neoadjuvant chemotherapy and RC with PLND is associated with the best long-term survival in patients with cN1 disease (62). The PLND template that extends to the level of the ureteric crossing is the standard approach because further extension does not improve oncological outcomes in terms of RFS, DFS, or OS (62).

Notably, the recent NIAGARA trial demonstrated that neoadjuvant chemotherapy plus perioperative durvalumab with RC significantly improved the estimated EFS and OS compared with neoadjuvant chemotherapy followed by RC in patients with MIBC (39). This study included a small proportion (5.5%; 58/1,063) of patients with cN1 disease, and subgroup analysis showed that the addition of perioperative durvalumab improved EFS in these patients (HR, 0.75; 95% CI, 0.33–1.64); the HR for OS was not calculated (39). These data suggest that the addition of perioperative durvalumab is worth considering in patients with cN1 disease, although its effects in this patient population should be further assessed in more dedicated studies.

Statement 2: Pelvic radiation can be considered in patients with cN1 disease who have no progression after systemic therapy.

A retrospective analysis (63) showed that RC (n = 76; 66% exposed to chemotherapy) and bladder-sparing TMT with radical-dose RT (n = 87; 80% exposed to chemotherapy) yielded similar OS (HR, 0.94; 95% CI, 0.63–1.41; P = 0.76) and PFS (HR, 0.74; 95% CI, 0.50–1.08; P = 0.12) in patients with cN+ UC (~70% with cN1 disease).

A larger retrospective study included 3,227 patients from the National Cancer Database in the USA who underwent multiagent systemic chemotherapy along with either high-intensity (RC + PLND or ≥ 50-Gy RT + TURBT) or conservative local treatment (TURBT alone, low-dose RT alone, or observation) for cN+ UC (cN1, 43%) (64). Overall, 784 (24.3%) and 2,443 (75.7%) patients received high-intensity and conservative local treatment, respectively (64). The high-intensity group had a significantly higher 5-year OS rate (28.4% vs. 18.3%; P < 0.001) than the conservative group (64). Within the high-intensity group, the RC and RT subgroups showed no significant difference in the 5-year OS rate (31.7% vs. 20.5%; P = 0.092) (64).

These data suggest that radical RT to the pelvis can be considered in patients with cN1 disease who have no progression after systemic therapy. However, there remains a lack of high-level evidence to support the use of bladder-sparing TMT for cN1 disease. The consensus among most panellists is that operable cN1 disease should primarily be treated with RC (± perioperative treatment).

Statement 3: For inoperable locally advanced UC, novel treatments, e.g. enfortumab vedotin + pembrolizumab (EV+P), may be considered.

The optimal care for inoperable cN1 disease remains undetermined. Most panellists considered the treatment approach for this disease entity comparable to that for M1 disease, i.e. upfront systemic therapy. The management of mUC was detailed in Part 3. Briefly, the feasibility of using EV+P for inoperable cN1 disease is extrapolated from the phase III randomised EV-302 trial with most patients having mUC (only 5% had locally advanced disease) that demonstrated survival benefits of EV+P over platinum-based chemotherapy (65). Other possible treatments for inoperable cN1 disease include pembrolizumab monotherapy for cisplatin-ineligible patients, especially those with high PD-L1 expression (derived from the phase II single-arm KEYNOTE-052 trial) (66), and cisplatin-based chemotherapy plus concurrent chemo-RT followed by maintenance avelumab (derived from two cases reported in Hong Kong) (67).

#### 3.1.6 Part 1.6 Follow-up and monitoring

Statement 1: After RC, patients should be followed up using CT of the thorax, abdomen, and pelvis (TAP) every 3–6 months for 2 years, then every 6–12 months for 3 years, and yearly thereafter.

Statement 2: After TMT, patients should be followed up using CT TAP, cystoscopy, and urine cytology every 3–6 months for 3 years and then every 6 months thereafter.

According to Chinese and Western guidelines (11, 51, 68), patients who receive RC and TMT have different risks of disease recurrence and thus require different monitoring strategies post-treatment. In general, compared with post-RC patients, post-TMT patients should be followed up more frequently, using cystoscopy and urine cytology on top of CT.

### 3.2 Part 2 – Management of locally advanced UTUC

**Figure 2:**
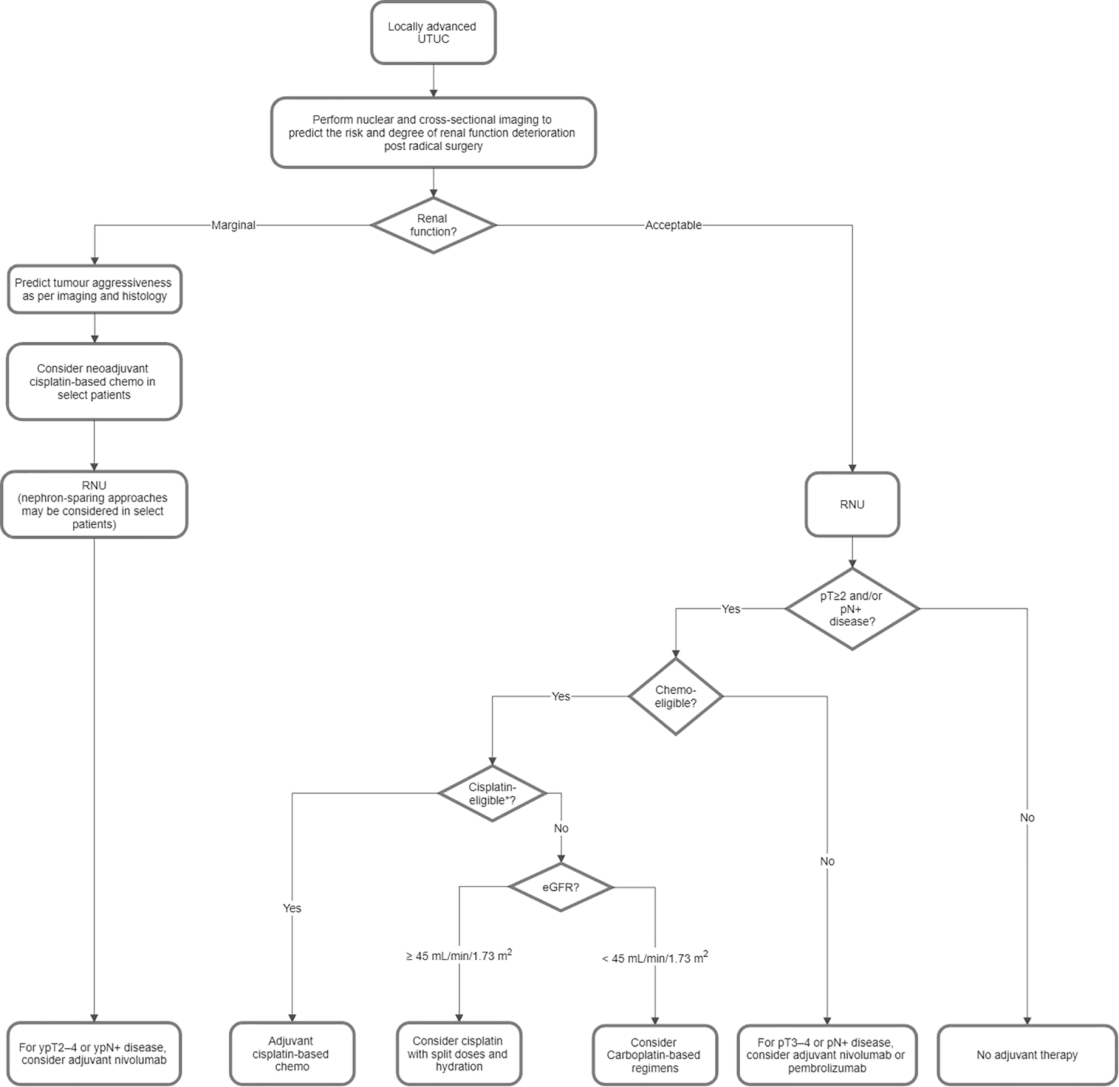
Proposed flowchart for the treatment of locally advanced upper tract urothelial carcinoma (UTUC). *Ineligibility for cisplatin is based on the Galsky criteria, i.e. ECOG PS 2 or Karnofsky PS < 60%–70%, CrCl < 60 mL/min, NYHA class III heart failure, grade ≥ 2 hearing loss, and/or grade ≥ 2 peripheral neuropathy. Chemo, chemotherapy; CrCl, creatinine clearance; ECOG, Eastern Cooperative Oncology Group; eGFR, estimated glomerular filtration rate; NYHA, New York Heart Association; PS, performance status; RNU, radical nephroureterectomy.

**Figure 2** shows a proposed flowchart for the treatment of locally advanced UTUC based on the consensus statements in Part 2.1.

#### 3.2.1 Part 2.1 Considerations for prescribing neoadjuvant or adjuvant platinum-based chemotherapy or IO in patients with UTUC

Statement 1: Adjuvant platinum-based chemotherapy after radical nephroureterectomy (RNU) should be offered to eligible patients with pT2–T4 and/or pN+ disease.

In the phase III randomised POUT study (69, 70), 261 patients with locally advanced UTUC (pT2–T4 and/or pN+) were randomised after RNU to surveillance or platinum-based chemotherapy (GemCis for glomerular filtration rate [GFR] ≥ 50 mL/min or gemcitabine–carboplatin [GemCarbo] for GFR < 50 mL/min) within 90 days post-surgery. The primary analysis (69) demonstrated that adjuvant platinum-based chemotherapy significantly improved the median DFS. Consistently, the final analysis (70) showed that the intervention was associated with a significantly higher 5-year DFS rate (62% vs. 45%; HR, 0.55; 95% CI, 0.38–0.80; P = 0.001) and a significantly higher 5-year OS rate (a secondary endpoint; 66% vs. 57%; HR, 0.68; 95% CI, 0.46–1.00; P = 0.049) compared with surveillance alone. The chemotherapy group had a 44% rate of acute grade ≥ 3 treatment-emergent adverse events (69). The choice of platinum did not impact the treatment effects (69, 70). In view of the DFS and OS benefits, adjuvant platinum-based chemotherapy should be offered to eligible patients who have undergone RNU for locally advanced UTUC.

Statement 2: Carboplatin-based regimens are not suggested in patients who are cisplatin-eligible.

Although there is a lack of randomised trials comparing cisplatin-based and carboplatin-based chemotherapy (71), cisplatin is generally anticipated to be more effective than carboplatin (72). The POUT final analysis revealed that, compared with the carboplatin subgroup, the cisplatin subgroup appeared to have a similar HR for disease recurrence or death (0.53 vs. 0.58) but a lower HR for death (0.57 vs. 0.87), suggesting a greater OS benefit, although the statistical interaction between the subgroups could not be properly assessed due to insufficient sample size (70). *In vitro* studies have shown that cisplatin may exert direct immunomodulatory effects on UC cells, possibly offering more durable anticancer activities than carboplatin (73). Taken together, the current evidence suggests that cisplatin-based chemotherapy should be offered to eligible patients, whereas carboplatin-based chemotherapy should only be considered for cisplatin-ineligible patients.

Statement 3: With split doses and hydration, cisplatin may be considered in patients with an estimated GFR (eGFR) down to 45 mL/min/1.73 m^2^.

A systematic review and network meta-analysis (74) included 1,767 patients on split-dose cisplatin (i.e. the standard cisplatin dose is administered over 2 days instead of 1 day) for locally advanced/mUC from a total of 16 mostly non-randomised or observational studies. Among these patients, the most common reason for using split-dose instead of standard-dose cisplatin was renal impairment (74). Although the included studies were heterogeneous, the overall findings demonstrated that split-dose cisplatin appeared to be comparable to standard-dose cisplatin and superior to carboplatin in terms of treatment response and survival outcomes (74). The EAU guidelines on UTUC (75) have suggested that split-dose cisplatin, with hydration, may be considered in patients with a creatinine clearance (CrCl) as low as 45 mL/min.

Most panellists noted that eGFR is preferred over CrCl as a measure of renal function in routine clinical practice. They decided that in patients with an eGFR of ≥ 45 to < 60 mL/min/1.73 m^2^ after RNU or during adjuvant standard-dose cisplatin-based chemotherapy, split-dose cisplatin, supplemented with hydration, may be considered to reduce nephrotoxicity while maximising anticancer effects.

Statement 4: There is currently no high-level evidence to support the use of neoadjuvant IO.

The PURE-02 feasibility study investigated neoadjuvant pembrolizumab monotherapy for high-risk UTUC (76). However, the major limitations of this study included a minimal sample size (only 10 patients) and, most importantly, failure to demonstrate the clinical benefits of the intervention (76). The nivolumab/ipilimumab combination as neoadjuvant IO for cisplatin-ineligible patients with UTUC is being investigated in an ongoing phase II study (77). The first stage results (77) showed that, of nine patients recruited, six received all planned treatments and had no disease progression; all patients underwent RNU, with pathological CR achieved in 3/9 patients (33%) and < ypT2pN0 in 6/9 patients (67%). The study has proceeded to the second stage. Further research is warranted to evaluate the clinical benefits of neoadjuvant IO for locally advanced UTUC.

Statement 5: Adjuvant nivolumab therapy can be considered in patients with ypT2–4 or ypN+ UTUC after neoadjuvant cisplatin-based chemotherapy (without IO).

Statement 6: Adjuvant nivolumab therapy can be considered in patients with pT3–4 or pN+ UTUC who have not received neoadjuvant cisplatin-based chemotherapy.

Statements 5 and 6 were derived from the phase III randomised CheckMate 274 study (46, 47), which investigated the use of adjuvant nivolumab in patients with muscle-invasive UC, with 21% of patients having a UTUC. Patients were eligible for the study regardless of whether they had received neoadjuvant chemotherapy. Extrapolated from the DFS and OS benefits of adjuvant nivolumab shown in the overall population, most panellists considered the intervention as a possible therapeutic option for patients with locally advanced UTUC, especially when a substantial proportion of patients are anticipated to be cisplatin-ineligible due to renal dysfunction post-RNU (74).

Statement 7: Adjuvant pembrolizumab therapy can be considered in patients with pT3–4 or pN+ UTUC who have not received neoadjuvant cisplatin-based chemotherapy.

In the phase III randomised AMBASSADOR study (48), adjuvant pembrolizumab significantly improved DFS, but not OS, in patients with muscle-invasive UC, with 22% of patients have a UTUC. Patients were eligible for the study regardless of whether they had received neoadjuvant chemotherapy. Regarding a draft statement on the use of adjuvant pembrolizumab in patients with locally advanced UTUC who *have received* neoadjuvant cisplatin-based chemotherapy, the panellists did not reach a consensus (only 75% accepted completely or with some reservations). In contrast, a consensus (92%) was reached for Statement 5 regarding the use of adjuvant nivolumab in such a patient population. Regarding patients who *have not received* neoadjuvant cisplatin-based chemotherapy, a higher level of consensus was reached for using adjuvant nivolumab than adjuvant pembrolizumab (100% vs. 83%). These results suggested that the panellists were generally more cautious about adjuvant pembrolizumab, primarily because of the lack of OS benefits. Referring to Statement 7, most panellists still considered adjuvant pembrolizumab as a worthwhile regimen to reduce the risk of disease recurrence in patients who have not received neoadjuvant chemotherapy; however, many of them thought that the overall benefit of the regimen might not be substantial in patients with prior neoadjuvant chemotherapy, and hence no consensus was reached for the pertinent draft statement.

Statement 8: There is currently no high-level evidence to support the use of perioperative RT.

Based on a systematic review and meta-analysis (78) of 20 studies, adjuvant RT may improve locoregional control, but not OS, in patients with locally advanced or margin-positive UTUC following RNU. Notably, most of the included studies were retrospective studies and highly heterogeneous. Currently, there is no high-level evidence to support neoadjuvant or adjuvant RT in patients with locally advanced UTUC.

Statement 9: Nuclear and cross-sectional imaging should be performed to help predict the risk and the degree of renal function deterioration after radical surgery.

Statement 10: Nephron-sparing approaches may be considered in patients with marginal renal function.

Statement 11: In selected patients with marginal renal function, prediction of tumour aggressiveness based on imaging and histology should be performed so that neoadjuvant chemotherapy can be considered for patients with a high-risk tumour.

Most panellists suggested the need to predict post-RNU renal function in patients with locally advanced UTUC. Nephron-sparing approaches, such as segmental ureterectomy, are anticipated to offer similar oncological outcomes and better preservation of renal function compared with RNU; therefore, these approaches are preferred to RNU in most patients with low-risk UTUC and selected patients with high-risk UTUC, especially those with marginal renal function (79).

Post-RNU renal function deterioration is often the major obstacle to using cisplatin in the adjuvant setting. Prediction of post-RNU renal function and tumour aggressiveness helps to determine whether neoadjuvant versus adjuvant chemotherapy is more suitable for a particular patient. Although not a standard of care, neoadjuvant chemotherapy remains practical and generally well tolerated (80), especially in patients with marginal renal function. The utility of neoadjuvant chemotherapy may be reflected in CheckMate 274 and AMBASSADOR; ~50% of participants in both studies received such a regimen (46, 48).

To predict post-RNU renal function decline, the ROBUUST Collaborative Group created a nomogram using data on 490 patients with non-metastatic UTUC from 17 institutions worldwide (81). The model was aimed at predicting eGFR < 50 mL/min/1.73 m^2^ at 3 months post-RNU, which was the definition of renal insufficiency for cisplatin (81). There were 361 patients with baseline eGFR > 50 mL/min/1.73 m^2^, of whom 226 from 10 USA centres were included for internal validation, and 135 from four centres outside of the USA were included for external validation (81). The nomogram suggested that older age (≥ 70 vs. < 70 years), a lower pre-operative eGFR, the absence (vs. presence) of hydroureteronephrosis, and a higher body mass index were risk factors for a lower predicted post-operative eGFR (81). The authors added that the operated kidney’s contribution to overall renal function could be more accurately assessed using nuclear renal scans or certain alternative factors, including tumour size, multifocality, ureteral location, and hydronephrosis (81). A study in Taiwan also showed that pre-operative hydronephrosis was associated with a lower decline in post-RNU renal function for UTUC (82). Based on these findings, the panellists suggested using nuclear and cross-sectional imaging to help predict post-RNU renal function decline.

Multiple retrospective studies have explored pre-operative predictors of tumour aggressiveness and prognosis that may facilitate the decision-making on the use of neoadjuvant cisplatin-based chemotherapy in patients with UTUC and marginal renal function. The significant predictors of non-organ confined UTUC include male sex, hydronephrosis, and tumours with CT-detected local invasion, multifocality, sessile architecture, variant histology, a high grade on ureteroscopic biopsy, or a high grade on urinary cytology (83–86).

Notably, neoadjuvant chemotherapy for UTUC should be considered on a case-by-case basis. The panellists discussed that the major concern about neoadjuvant chemotherapy is the potential for chemotoxicity or disease progression due to suboptimal chemo-response, which can undermine the patient’s fitness for surgery. Several panellists suggested that neoadjuvant chemotherapy can be considered in patients with locally advanced disease and borderline renal function; however, patient selection criteria for neoadjuvant chemotherapy should be further optimised.

#### 3.2.2 Part 2.2 Optimal follow-up schedule in patients with UTUC

Statement 1: For high-risk tumours post-RNU, cross-sectional imaging of the abdomen and pelvis, preferably with CT urography, should be performed every 6–12 months for years 1–2, and then annually for years 3–4.

Statement 2: Chest imaging, preferably with CT of the thorax, should be performed every 6–12 months for the first 3–4 years.

Statement 3: The following can be considered as risk factors that may prompt more stringent follow-up schedules:

a. History of nephron-sparing surgery;
b. Smoking; or
c. Non-UC histology variant.

In patients who undergo RNU for high-risk UTUC, the risk of distant metastases or non-bladder recurrence appears to be the highest within the first 1–3 years post-surgery, gradually diminishing in the following years (75, 87, 88). The panellists considered that patients with high-risk UTUC should generally receive more frequent screening (i.e. every 6–12 months) within the first 1–3 years post-surgery. They also suggested that more stringent follow-up schedules can be considered to address the potentially elevated risks of metastases and disease recurrence in patients who undergo nephron-sparing surgery instead of RNU, patients who are smokers, and patients with micropapillary, squamous, glandular, or sarcomatoid histological variants (88, 89).

### 3.3 Part 3 – Management of unresectable locally advanced/mUC

**Figure 3:**
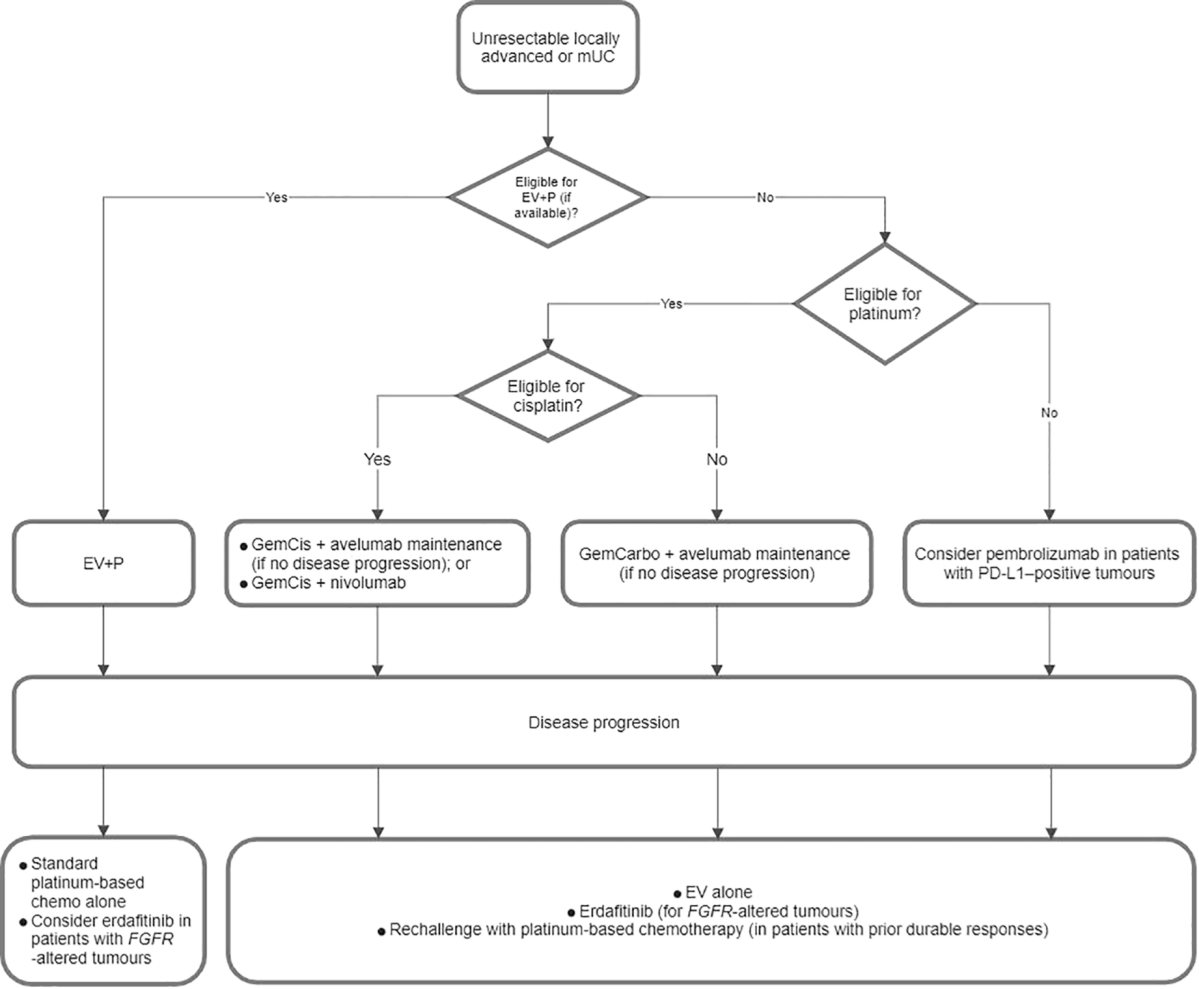
Proposed flowchart for the treatment of unresectable locally advanced or metastatic urothelial carcinoma (mUC). EV+P, enfortumab vedotin plus pembrolizumab; GemCarbo, gemcitabine plus carboplatin; GemCis, gemcitabine plus cisplatin; PD-L1, programmed death-ligand 1.

**Figure 3** demonstrates a proposed treatment algorithm of unresectable locally advanced/mUC that was developed from the consensus statements in Parts 3.1 and 3.2.

#### 3.3.1 Part 3.1 Initial treatment choice

Statement 1: EV+P is the preferred treatment regimen over platinum-based chemotherapy in eligible patients.

In the open-label randomised EV-302 trial (65) involving 886 patients with previously untreated locally advanced/mUC, treatment with EV+P significantly improved OS (median, 31.5 vs. 16.1 months; HR, 0.47; 95% CI, 0.38–0.58; P < 0.001) compared with platinum-based chemotherapy (GemCis or GemCarbo). The OS benefit was consistent across clinically relevant subgroups and was observed irrespective of age, sex, cisplatin eligibility, PD-L1 expression, and sites of metastases. The PFS (median, 12.5 vs. 6.3 months; HR, 0.45; 95% CI, 0.38–0.54; P < 0.001) and objective response rate (ORR; 67.7% vs. 44.4%; P < 0.001) were also significantly improved with EV+P, with 29.1% of patients on EV+P achieving CR, compared with only 12.5% in the chemotherapy arm. EV+P has been recommended as the preferred first-line treatment for locally advanced/mUC irrespective of platinum eligibility by multiple international guidelines, including the NCCN, the ESMO, and the EAU (11, 51, 90). Notably, treatment costs and health policies may be among the potential barriers to accessing EV+P, considering its novelty. Furthermore, caution should be exercised when EV+P is used in patients with pre-existing peripheral neuropathy or uncontrolled diabetes (65).

Statement 2: Preferred treatment options for cisplatin-eligible patients if EV+P is unavailable or contraindicated include:

a. GemCis chemotherapy and, if there is no disease progression, followed by avelumab maintenance; or
b. GemCis chemotherapy + nivolumab.

Statement 3: The preferred treatment option for cisplatin-ineligible patients is GemCarbo chemotherapy and, if there is no disease progression, followed by avelumab maintenance if EV+P is unavailable or contraindicated.

Eligibility for platinum-based regimens is an important factor for treatment decision-making in mUC. The EAU has suggested a set of criteria for assessing the eligibility for cisplatin and carboplatin (**Table 2**) (11), which are widely adopted in the clinical setting.

**Table 2:** Definitions of platinum-eligibility for first-line treatment of metastatic urothelial carcinoma (11).

Platinum-eligible		Platinum-ineligible
Cisplatin-eligible	Carboplatin-eligible	
All of the following should be fulfilled: • ECOG PS 0–1; • GFR > 50–60 mL/min; • Audiometric hearing loss grade < 2; • Peripheral neuropathy grade < 2; and • Cardiac insufficiency NYHA class < III	• ECOG PS 2 or GFR 30–60 mL/min; or • Not fulfilling other cisplatin-eligibility criteria	Any of the following: • ECOG PS > 2; • GFR < 30 mL/min; • ECOG PS 2 and GFR < 60 mL/min; or • Comorbidities > grade 2

ECOG, Eastern Cooperative Oncology Group; GFR, glomerular filtration rate; NYHA, New York Heart Association; PS, performance status.

According to a physician survey in European countries, 76.8% of patients with mUC were considered platinum-eligible (91). Other studies reported that up to 50% of patients with advanced UC were ineligible for cisplatin, primarily because of renal impairment (92); however, most of these patients may still be eligible for carboplatin.

Platinum-based chemotherapy for mUC generally yields an ORR of 40–50% and a disease control rate of 75–80%, and cisplatin-based regimens appear to offer a longer median OS (14–15 months vs. 9–10 months) compared with carboplatin-based regimens; however, most patients with mUC have disease progression within ~9 months after platinum-based chemotherapy (93–96).

The phase III randomised JAVELIN Bladder 100 trial demonstrated that, among patients with advanced or mUC who did not have disease progression with first-line platinum-based chemotherapy (4–6 cycles of GemCis or GemCarbo), the addition of maintenance avelumab to best supportive care (BSC) significantly improved median OS (21.4 vs. 14.3 months; HR, 0.69; 95% CI, 0.56–0.86; P = 0.001) compared with BSC alone (97). The OS benefit of maintenance avelumab remained (HR, 0.76; 95% CI, 0.63–0.91; two-sided P = 0.0036) after ≥ 2 years of follow-up (98). The first-line platinum-based chemotherapy duration (4 vs. > 4 cycles) and the interval before maintenance (4–< 6 vs. 6–< 8 vs. 8–10 weeks) did not affect the efficacy of maintenance avelumab (99). Additionally, the efficacy and safety of maintenance avelumab were consistent across different subgroups, including Asian patients (100), patients with high body mass index (101), and older patients (age ≥ 65 years) (102). Exploratory analyses of patient-reported outcomes showed that the JAVELIN participants on maintenance avelumab for ≥ 12 months may have experienced preservation of health-related quality of life and control of cancer-related symptoms with manageable treatment-related toxicity (103). GemCis or GemCarbo followed by avelumab maintenance if no disease progression has been recommended by several international guidelines (11, 51, 90).

In the phase III randomised CheckMate 901 trial (104), among cisplatin-eligible patients with previously untreated unresectable or mUC, treatment with nivolumab plus GemCis for ≤ 6 cycles, followed by nivolumab alone for ≤ 2 years, significantly improved OS (median, 21.7 vs. 18.9 months; HR, 0.78; 95% CI, 0.63–0.96; P = 0.02) and progression-free survival (PFS, median, 7.9 vs. 7.6 months; HR, 0.72; 95% CI, 0.59–0.88; P = 0.001) compared with GemCis for ≤ 6 cycles.

Nivolumab–chemotherapy combination is the only immunochemotherapy that demonstrates survival benefits versus chemotherapy alone in patients with mUC. Atezolizumab–chemotherapy was investigated but failed to show OS benefits compared with chemotherapy alone in the phase III randomised IMvigor130 trial (105, 106). Different from CheckMate 901, IMvigor130 allowed GemCis or GemCarbo in both treatment arms. Further analysis revealed that cisplatin ± atezolizumab was associated with improved survival outcomes compared with carboplatin ± atezolizumab, particularly in patients with tumours exhibiting preexisting adaptive immunity (73). One possible reason for these observations is that, compared with carboplatin, cisplatin has direct immunomodulatory effects on cancer cells *in vitro*, enhancing antigen-specific T-cell killing (73). Regardless, the atezolizumab–chemotherapy combination is not indicated for the treatment of mUC.

Separately, the panellists discussed that split-dose cisplatin is not recommended for cisplatin-ineligible patients because, despite possibly offering survival benefits, it may be associated with more frequent grade 4 neutropenia compared with GemCarbo in these patients (74, 107).

Statement 4: In patients who received prior adjuvant IO, the clinical benefits of IO-containing regimens as first-line treatment for mUC remain undetermined.

Adjuvant IO is a relatively novel treatment approach for MIBC. No prospective trials have investigated the efficacy and safety of IO-containing regimens, e.g. EV+P, in patients with newly diagnosed mUC who had prior exposure to adjuvant IO. Statement 4 was adapted from a recommendation from the EAU (11).

Statement 5: If EV+P is unavailable or contraindicated, pembrolizumab can be considered for platinum-ineligible patients with PD-L1–positive tumours.

The single-arm phase II KEYNOTE-052 study demonstrated that first-line pembrolizumab monotherapy conferred an ORR of 28.6% and a median OS of 11.3 months in patients with locally advanced or metastatic, cisplatin-ineligible UC (66). Notably, patients with positive tumour PD-L1 expression (i.e. combined positive score [CPS] ≥ 10) or LN-only disease had more favourable treatment outcomes with pembrolizumab (ORRs, 47.3% and 49.0%; median OS, 18.5 months and 27.0 months, respectively) (66). The European Medicines Agency has authorised pembrolizumab as a first-line treatment for cisplatin-unfit patients with locally advanced/mUC and positive PD-L1 expression (CPS ≥ 10). The U.S. Food and Drug Administration (FDA) similarly approved pembrolizumab for cisplatin-unfit patients, without regard for the PD-L1 status (108, 109).

#### 3.3.2 Part 3.2 Subsequent treatment approach

Statement 1: For patients who progress on EV+P, standard platinum-based chemotherapy without maintenance IO should be considered.

Statement 2: For patients who progress on EV+P and have *FGFR*-altered tumours, erdafitinib may be considered.

EV+P is a relatively novel first-line treatment approach for mUC; therefore, the optimal subsequent treatment for patients who progress on this regimen remains largely undetermined. However, data from the EV-302 trial may provide some insights. In the EV+P arm, 140/442 patients received subsequent anticancer therapies, most of whom (110/140) received platinum-based chemotherapy as second-line therapy; only seven patients received PD-1/L1 inhibitor-containing therapy (65). Based on these data, the panellists extrapolated that standard platinum-based chemotherapy without maintenance IO should be considered for patients who progress on EV+P.

The phase III randomised THOR study Cohort 1 demonstrated that, compared with chemotherapy (docetaxel or vinflunine), erdafitinib significantly improved median OS (12.1 vs. 7.8 months; HR, 0.64; 95% CI, 0.47–0.88; P = 0.005) in patients with mUC with susceptible *FGFR3/2* alterations who progressed on one or two prior therapies that included a PD-1/L1 inhibitor (110). Indeed, most of the study participants had received prior platinum-based chemotherapy, and none of them had received EV. However, most panellists agreed that, considering the lack of treatment options for later-line mUC, erdafitinib remains a possible treatment regimen for patients who progress on EV+P and have *FGFR3/2* alterations.

Statement 3: For patients who progress on platinum-based chemotherapy and avelumab maintenance, subsequent-line treatment options include EV, erdafitinib (for *FGFR*-altered tumours), or chemotherapy.

In the phase III open-label EV-301 trial (111), EV monotherapy significantly improved median OS (12.88 vs. 8.97 months; HR, 0.70; 95% CI, 0.56–0.89; P = 0.001) in patients with locally advanced/mUC who had received platinum-based chemotherapy and experienced disease progression during or after PD-1/L1 inhibitor therapy compared with standard chemotherapy (docetaxel, paclitaxel, or vinflunine). Exploratory analyses of the ongoing, real-world, ambispective AVENANCE study (112) showed that patients who received first-line platinum-based chemotherapy followed by maintenance avelumab, and received an antibody–drug conjugate (e.g. EV) as a second-line therapy, may have a median OS of > 3.5 years from the start of platinum-based chemotherapy. These data support EV as a subsequent treatment for patients who progress on platinum-based chemotherapy and avelumab maintenance.

Based on the aforementioned results from the THOR study Cohort 1 (110), erdafitinib is another feasible treatment regimen for this patient population, given that they carry *FGFR3/2* alterations.

The ESMO guidelines have suggested taxane chemotherapy as a treatment regimen for patients who progress on platinum-based chemotherapy and a PD-1/L1 inhibitor, although the evidence is not robust (90).

A systematic review and meta-analysis revealed that, despite significantly improving ORR and PFS, doublet chemotherapy (taxane plus cisplatin or carboplatin) did not prolong OS compared with single-agent chemotherapy (vinflunine, paclitaxel, or docetaxel) as second-line therapy for patients who progressed on platinum-based chemotherapy (113).

Statement 4: In patients who had prior durable responses to platinum-based regimens, rechallenge with platinum-based chemotherapy may be considered.

Wong et al. analysed data from the Retrospective International Study of Cancers of the Urothelium to compare clinical outcomes of platinum‐based chemotherapy versus non‐platinum–based chemotherapy as subsequent treatment for patients who received first‐line platinum‐based chemotherapy for mUC (114). Rechallenge with platinum-based chemotherapy was associated with better OS and disease control than subsequent non-platinum–based chemotherapy. However, it should be noted that, among patients who were rechallenged with platinum-based chemotherapy, those who achieved disease control were more likely to have no liver metastases, have achieved disease control with first-line platinum-based chemotherapy, and have a longer median elapsed time since first-line platinum-based chemotherapy (6.0 vs. 2.9 months; P = 0.008).

In a multicentre PSM study of patients who received platinum-based chemotherapy and pembrolizumab for advanced UC (115), subsequent treatment with EV (n = 39) or platinum rechallenge (n = 25) yielded similar median PFS (5 vs. 8 months) and OS (11 vs. 12 months).

These data suggest that platinum rechallenge could be a treatment regimen for selected patients who progress on first-line platinum-based chemotherapy ± PD-1/L1 inhibitor therapy, especially if they have achieved disease control and durable response with initial platinum-based chemotherapy.

Statement 5: For patients who progress after IO, rechallenge with a PD-1 or PD-L1 inhibitor is not recommended.

The investigational arms of the EV-302 (65), JAVELIN Bladder 100 (97), and CheckMate 901 trials (104) included participants who received PD-1/L1 inhibitors in the first-line setting. Data from these studies showed that subsequent treatment with a PD-1/L1 inhibitor was only used in a minimal number of these participants when they experienced disease progression. There is a lack of evidence on the clinical outcomes of rechallenging with IO in IO-experienced patients with mUC.

Statement 6: The choice of subsequent therapy should be individualised based on patient performance status, prior treatment responses and tolerability, and biomarker status.

The panellists discussed the potential for pembrolizumab, vinflunine, or sacituzumab govitecan (SG) as a subsequent treatment for patients with mUC who progress on first-line platinum-based chemotherapy ± PD-1/L1 inhibitor therapy.

The phase III open-label KEYNOTE-045 trial of patients with advanced UC that recurred or progressed after platinum-based chemotherapy demonstrated that pembrolizumab monotherapy significantly improved median OS in the total population (10.3 vs. 7.4 months; HR, 0.73; 95% CI, 0.59–0.91; P = 0.002) and in patients who had a PD-L1–positive (CPS ≥ 10) tumour (8.0 vs. 5.2 months; HR, 0.57; 95% CI, 0.37–0.88; P = 0.005) compared with the investigator’s choice of chemotherapy with paclitaxel, docetaxel, or vinflunine (116). However, pembrolizumab did not significantly improve PFS compared with chemotherapy, regardless of PD-L1 status (116). The ESMO has recommended pembrolizumab as a treatment regimen for patients with platinum-refractory mUC who have no prior exposure to PD-1/L1 inhibitors (90).

In a phase III open-label randomised trial of patients with mUC who progressed on first-line platinum-based chemotherapy, vinflunine plus BSC significantly improved median OS (6.9 vs. 4.3 months; P = 0.040) after adjusting for protocol violations compared with BSC alone (117). In the ESMO guidelines, vinflunine is a treatment option for patients who progress on platinum-based chemotherapy and a PD-1/L1 inhibitor (90).

The use of SG for the treatment of mUC is debatable. The open-label phase II TROPHY-U-01 study showed that, in the cohort of 113 patients with locally advanced or mUC who progressed after platinum-based chemotherapy and IO, subsequent SG treatment yielded an ORR of 28%, a median PFS of 5.4 months, and a median OS of 10.9 months (118). Based on these results, the FDA granted accelerated approval for SG as a treatment regimen for the captioned patient population (119). However, the randomised phase III TROPiCS-04 trial showed that SG did not significantly improve median OS in a similar patient population compared with standard chemotherapy (paclitaxel, docetaxel, or vinflunine), meaning that the primary endpoint was missed (120, 121). Subsequently, the manufacturer decided to voluntarily withdraw the FDA indication for SG in mUC (119). Currently, the ESMO still lists SG as a treatment option for mUC in the post-platinum, post-IO setting (90).

#### 3.3.3 Part 3.3 Management of oligometastatic BC

Statement 1: Oligometastatic BC (OMBC) is defined as having ≤ 3 metastatic sites that are resectable or amenable to stereotactic therapy.

OMBC is an intermediate entity between localised cancer and extensively metastatic disease (122). Researchers have an emerging interest in determining the optimal treatment approach for OMBC, which likely involves the combination of systemic and local therapies (122). Relevant studies mostly regarded OMBC as having up to 3 or 5 metastatic sites that were amenable to local therapy (surgery or irradiation) (123). Additionally, most cases were *de novo* OMBC (initial diagnosis of primary BC with limited metastases), but OMBC also included metachronous oligorecurrence (diagnosis of limited metastases after treatment of localised BC) and metachronous oligoprogression (progression of a limited number of metastatic lesions while others are controlled with active systemic therapy) (123).

Statement 2: Systemic therapy remains the mainstay of treatment for OMBC.

Most studies reported that patients with OMBC received systemic treatment, primarily platinum-based chemotherapy, followed by metastasis-directed therapy, which was considered more beneficial in patients with favourable responses to systemic therapy (123). Multiple studies are investigating the use of IO (e.g. PD-1/L1 inhibitors) plus stereotactic body RT (SBRT) as local therapy for OMBC (124, 125). The results of these studies will further inform the treatment paradigm for OMBC.

Statement 3: SBRT to the metastatic site may be considered in patients with OMBC.

An analysis included 61 patients who received SBRT ± concomitant systemic therapy for the management of OMBC (60.4% of patients had 1 metastatic site; 39.3% had 2–5 metastatic sites) from three institutions (126). Among a total of 82 lesions treated with SBRT (median biologically effective dose [BED10], 78.7 Gy), 40.2% and 35.4% were in the lungs and LNs, respectively (126). With a median follow-up of 17.2 months, rates of local control at 1 and 2 years were 92% and 88.9%, respectively (126). The univariate analysis revealed that lines of systemic therapy before SBRT were associated with inferior local control (HR, 2.62; 95% CI, 1.07–6.40; P = 0.034) (126). The median overall PFS was 10.1 months and negatively correlated with the number of metastases (HR, 2.65, 95% CI, 1.29–5.44; P = 0.008) (126). The median OS was 25.6 months and positively correlated with the total SBRT dose (HR, 0.93; 95% CI, 0.90–0.97; P = 0.003) and the BED10 (HR, 0.97; 95% CI, 0.96–0.99; P = 0.006) (126). No grade ≥ 2 adverse events were reported (126). Other retrospective studies also demonstrated that metastasis-directed RT with consolidative intent during or after systemic therapy conferred promising OS and PFS benefits, with an acceptable toxicity profile, in patients with OMBC (≤ 5 metastatic sites) (127, 128). The ongoing randomised phase II BLAD-RAD01 trial will further investigate the role of local consolidative RT in patients who receive platinum-based chemotherapy (plus maintenance avelumab in progression-free patients) for OMBC (≤ 3 metastatic sites) (129).

Statement 4: Metastasectomy may be considered in highly selected patients with OMBC.

Besides SBRT, metastasectomy is another feasible metastasis-directed approach for the treatment of oligometastatic disease (130). However, the clinical outcomes of metastasectomy in patients with OMBC are inconsistent. In a single-centre retrospective study of 22 patients who were treated with metastasectomy for oligorecurrent UC in a single organ with ≤ 3 metastases (mostly in the lungs), the 5-year OS, CSS, and secondary RFS rates were 51.4%, 57.0%, and 49.9%, respectively (131). Primary UTUC involvement, hepatic metastasectomy, size of the largest lesion > 8 mm, and the presence of > 1 pulmonary lesion were associated with poorer survival outcomes (131). An analysis of the US National Cancer Database revealed that metastasectomy was performed in 7% of patients with mUC, most of whom were younger, had > cT3 disease, and had received radical surgery to the primary tumour and systemic therapy (132). However, metastasectomy was not associated with an OS benefit (HR, 0.94; 95% CI, 0.83–1.07; P = 0.38) after PSM (132). Taken together, these data suggest that metastasectomy may only benefit a highly selected group of patients with OMBC, such as those with a minimal number of small lesions confined in the lungs.

## 4 Discussion and conclusion

These consensus statements aim to optimise the care for patients with advanced UC, including MIBC, locally advanced UTUC, and inoperable locally advanced/mUC, who generally have a poor prognosis and lowered quality of life (3). The advantages of these statements include the consideration of evidence on state-of-the-art therapeutic agents (e.g. ICIs and antibody–drug conjugates), surgical approaches (e.g. *en bloc* resection and nephron-sparing surgery) and RT techniques (e.g. image guidance, hypofractionation, and SBRT), in addition to expert insights into optimal treatment and monitoring strategies. However, several caveats of these statements should be noted. First, rapid developments in novel medications and an ever-changing treatment landscape of advanced UC may prompt frequent updates to these statements. Second, regarding the treatment of locally advanced UTUC, patient selection criteria for neoadjuvant chemotherapy remain to be further optimised. Third, treatment costs and healthcare systems’ protocols may be among the barriers to accessing advanced treatment modalities. Furthermore, the panel did not reach a consensus on four suggestions: 1) considering incomplete resection one ineligibility criterion for TMT in patients with MIBC; 2) considering adjuvant pembrolizumab in patients with prior neoadjuvant chemotherapy for UTUC; and considering 3) metabolic syndrome or 4) obesity as risk factors that may prompt more stringent follow-up schedules in patients with UTUC post-surgery. The current evidence appears to be unsupportive or inconclusive regarding these suggestions, underscoring the need for further investigations.

In conclusion, the panel anticipates that these statements could serve as a practical recommendation for clinicians in Hong Kong and possibly the Asia-Pacific region, where similar guidelines or recommendations are limited, regarding the management of patients with advanced UC.
