# Supplementary material for: Consensus statements from the Hong Kong Urological Association and the Hong Kong Society of Uro-Oncology on the management of muscle-invasive and advanced urothelial carcinoma
Source: Front Oncol. 2025 May 8;15:1564487. doi: 10.3389/fonc.2025.1564487 (PMC12095001; doi:10.3389/fonc.2025.1564487)
Supplement: Supplementary file 1 [file Table1.docx]

Appendix S1. Full voting records for all accepted and rejected statements.

**Part 1 – Management of non-metastatic muscle-invasive and locally advanced urothelial carcinoma (UC) of the bladder**

| **1.1. Candidacy for radical cystectomy (RC), radiotherapy (RT), or trimodal bladder-sparing therapy (TMT)** | | **Response options* (%)** | | | | | **Statement was accepted only if (A + B)% ≥ 80%** | | | | |
| --- | --- | --- | --- | --- | --- | --- | --- | --- | --- | --- | --- |
| **#** | **Drafted statements** | **A** | **B** | **C** | **D** | **E** | **A + B (%)** | **Accepted** | | **Voted down** | |
| **1** | RC with standard pelvic lymph node dissection is the standard treatment for patients with non-metastatic muscle-invasive bladder cancer (MIBC). | 83.33 | 16.67 | 0 | 0 | 0 | 100 | ✓ | |  | |
| **2** | For male patients, cystectomy with sexual-preserving techniques should only be considered in patients with organ-confined disease and in those without tumours at the prostate, prostatic urethra, or bladder neck. | 83.33 | 16.67 | 0 | 0 | 0 | 100 | ✓ | |  | |
| **3** | For female patients, sexual organ-preserving cystectomy should only be considered in patients with organ-confined disease. | 58.33 | 41.67 | 0 | 0 | 0 | 100 | ✓ | |  | |
| **4** | Patients who want to preserve sexual function should be counselled on various sexual-preserving surgical techniques. | 75 | 25 | 0 | 0 | 0 | 100 | ✓ | |  | |
| **5** | A positive prostatic urethral biopsy in transurethral resection of bladder tumour (TURBT) does not correlate with the final margin and should not exclude patients from consideration for orthotopic bladder reconstruction. | 41.67 | 41.67 | 8.33 | 8.33 | 0 | 83.33 | ✓ | |  | |
| **6** | Before undergoing RC, the Enhanced Recovery After Surgery (ERAS) approach should be considered to reduce the risks of post-operative ileus and venous thromboembolism. | 75 | 25 | 0 | 0 | 0 | 100 | ✓ | |  | |
| **7a** | To decide whether a patient is fit for RC, the following factor should be considered: Age | 50 | 50 | 0 | 0 | 0 | 100 | ✓ | |  | |
| **7b** | To decide whether a patient is fit for RC, the following factor should be considered: Performance status (e.g. Eastern Cooperative Oncology Group) | 100 | 0 | 0 | 0 | 0 | 100 | ✓ | |  | |
| **7c** | To decide whether a patient is fit for RC, the following factor should be considered: Cardiorespiratory function | 91.67 | 8.33 | 0 | 0 | 0 | 100 | ✓ | |  | |
| **7d** | To decide whether a patient is fit for RC, the following factor should be considered: Comorbidities (e.g. Charlson index) | 83.33 | 16.67 | 0 | 0 | 0 | 100 | ✓ | |  | |
| **7e** | To decide whether a patient is fit for RC, the following factor should be considered: Frailty | 83.33 | 16.67 | 0 | 0 | 0 | 100 | ✓ | |  | |
| **7f** | To decide whether a patient is fit for RC, the following factor should be considered: Cognitive function | 41.67 | 50 | 8.33 | 0 | 0 | 91.67 | ✓ | |  | |
| **8** | RT alone should only be a treatment option for patients who are unfit for both RC and concurrent chemo-irradiation. | 83.33 | 16.67 | 0 | 0 | 0 | 100 | ✓ | |  | |
| **9** | TMT should include all three modalities, i.e. maximal TURBT, radiosensitising agents (e.g. chemotherapy), and RT. | 83.33 | 16.67 | 0 | 0 | 0 | 100 | ✓ | |  | |
| **10a** | TMT is NOT preferred in patients with the following characteristic: Multiple tumours | 50 | 41.67 | 0 | 8.33 | 0 | 91.67 | ✓ | |  | |
| **10b** | TMT is NOT preferred in patients with the following characteristic: Incomplete resection | 33.33 | 41.67 | 16.67 | 8.33 | 0 | 75 |  | | X | |
| **10c** | TMT is NOT preferred in patients with the following characteristic: cT3 or above tumours | 75 | 16.67 | 0 | 8.33 | 0 | 91.67 | ✓ | |  | |
| **10d** | TMT is NOT preferred in patients with the following characteristic: Presence of extensive or multifocal carcinoma *in situ* | 75 | 16.67 | 0 | 8.33 | 0 | 91.67 | ✓ | |  | |
| **10e** | TMT is NOT preferred in patients with the following characteristic: Presence of tumour-related hydronephrosis | 66.67 | 25 | 8.33 | 0 | 0 | 91.67 | ✓ | |  | |
| **10f** | TMT is NOT preferred in patients with the following characteristic: Suboptimal pre-treatment bladder function | 58.33 | 33.33 | 8.33 | 0 | 0 | 91.67 | ✓ | |  | |
| **10g** | TMT is NOT preferred in patients with the following characteristic: Poor patient compliance with lifelong bladder monitoring | 58.33 | 41.67 | 0 | 0 | 0 | 100 | ✓ | |  | |
| **11** | Magnetic resonance imaging (MRI) is the preferred imaging technique for staging the primary tumour. | 41.67 | 50 | 8.33 | 0 | 0 | 91.67 | ✓ | |  | |
| **12** | Contrast computed tomography (CT) of the chest, abdomen, and pelvis should be considered for the screening of nodal and distant metastases. | 66.67 | 33.33 | 0 | 0 | 0 | 100 | ✓ | |  | |
| **13** | CT urography should be considered to evaluate the presence of any upper tract UC (UTUC). | 83.33 | 16.67 | 0 | 0 | 0 | 100 | ✓ | |  | |
| **14** | Positron emission tomography-CT can be considered for the screening of nodal and distant metastases. | 66.67 | 33.33 | 0 | 0 | 0 | 100 | ✓ | |  | |
| **15** | Contrast MRI of the abdomen and pelvis is a suitable alternative for the screening of lymph node and visceral metastases, and MR urography for the detection of UTUC. | 33.33 | 66.67 | 0 | 0 | 0 | 100 | ✓ | |  | |
| **16** | Routine screening for asymptomatic brain or bone metastases is not recommended. | 66.67 | 33.33 | 0 | 0 | 0 | 100 | ✓ | |  | |
| **Total:** | | | | | | | | | 26 | | 1 |

*Response options include A: accept completely; B: accept with some reservation; C: accept with major reservation; D: reject with reservation; and E: reject completely.

| **1.2. Role of neoadjuvant and adjuvant systemic pharmacotherapy** | | **Response options* (%)** | | | | | | **Statement was accepted only if (A + B)% ≥ 80%** | | | | | | |
| --- | --- | --- | --- | --- | --- | --- | --- | --- | --- | --- | --- | --- | --- | --- |
| **#** | **Drafted statements** | **A** | **B** | **C** | **D** | **E** | **A + B (%)** | | **Accepted** | | **Voted down** | | | |
| **1** | In patients with cT2–4a N0M0 disease who will undergo RC and are cisplatin-eligible, the option of neoadjuvant cisplatin-based chemotherapy plus perioperative durvalumab immunotherapy (IO) should be offered. | 66.67 | 33.33 | 0 | 0 | 0 | 100 | | ✓ | | |  | | |
| **2** | There is currently a lack of level I evidence to support the use of IO, without chemotherapy, in the neoadjuvant setting. | 91.67 | 8.33 | 0 | 0 | 0 | 100 | | ✓ | | |  | | |
| **3** | Adjuvant chemotherapy should only be considered in patients who have not received neoadjuvant chemotherapy and have advanced disease, i.e. pT3/4 and/or pN+ disease. | 75 | 16.67 | 8.33 | 0 | 0 | 91.67 | | ✓ | | |  | | |
| **4** | Adjuvant nivolumab therapy can be considered in patients with ypT2–4a or ypN+ MIBC after neoadjuvant cisplatin-based chemotherapy (without IO). | 75 | 25 | 0 | 0 | 0 | 100 | | ✓ | | |  | | |
| **5** | Adjuvant pembrolizumab therapy can be considered in patients with ypT2–4a or ypN+ MIBC after neoadjuvant cisplatin-based chemotherapy (without IO). | 66.67 | 16.67 | 16.67 | 0 | 0 | 83.33 | | ✓ | | |  | | |
| **6** | Adjuvant nivolumab therapy can be considered in patients with pT3–4a or pN+ MIBC who have not received neoadjuvant cisplatin-based chemotherapy. | 66.67 | 33.33 | 0 | 0 | 0 | 100 | | ✓ | | |  | | |
| **7** | Adjuvant pembrolizumab therapy can be considered in patients with pT3–4a or pN+ MIBC who have not received neoadjuvant cisplatin-based chemotherapy. | 58.33 | 25 | 16.67 | 0 | 0 | 83.33 | | ✓ | | |  | | |
| **Total:** | | | | | | | | | | 7 | | | 0 |  |

*Response options include A: accept completely; B: accept with some reservation; C: accept with major reservation; D: reject with reservation; and E: reject completely.

| **1.3. Role of RT in MIBC** | | **Response options* (%)** | | | | | **Statement was accepted only if (A + B)% ≥ 80%** | | | | |  |  |
| --- | --- | --- | --- | --- | --- | --- | --- | --- | --- | --- | --- | --- | --- |
| **#** | **Drafted statements** | **A** | **B** | **C** | **D** | **E** | **A + B (%)** | **Accepted** | | **Voted down** | | |  |
| **1** | When TMT is considered, image-guided hypofractionated RT to the whole bladder concurrent with radiosensitising agents should be the standard of care. | 66.67 | 25 | 8.33 | 0 | 0 | 91.67 | ✓ | |  | | |  |
| **2** | Radiosensitising agents (e.g. fluorouracil/mitomycin, cisplatin, low-dose gemcitabine, etc.) should be concurrently given with RT in TMT. | 91.67 | 8.33 | 0 | 0 | 0 | 100 | ✓ | |  | | |  |
| **3** | There is currently a lack of evidence to support the use of adjuvant RT after RC. | 75 | 16.67 | 8.33 | 0 | 0 | 91.67 | ✓ | |  | | |  |
| **Total:** | | | | | | | | | 3 | | 0 | | |

*Response options include A: accept completely; B: accept with some reservation; C: accept with major reservation; D: reject with reservation; and E: reject completely.

| **1.4. Surgical aspects of TMT** | | **Response options* (%)** | | | | | **Statement was accepted only if (A + B)% ≥ 80%** | | | | |  |  |
| --- | --- | --- | --- | --- | --- | --- | --- | --- | --- | --- | --- | --- | --- |
| **#** | **Drafted statements** | **A** | **B** | **C** | **D** | **E** | **A + B (%)** | **Accepted** | | **Voted down** | | |  |
| **1** | If there is a suboptimal response to TMT, salvage cystectomy should be considered. | 83.33 | 16.67 | 0 | 0 | 0 | 100 | ✓ | |  | | |  |
| **2** | Modified *en bloc* resection may be feasible in TMT for MIBC, depending on MRI findings. | 41.67 | 50 | 8.33 | 0.00 | 0 | 91.67 | ✓ | |  | | |  |
| **3** | Second-look TURBT is recommended in TMT. | 66.67 | 25 | 8.33 | 0 | 0 | 91.67 | ✓ | |  | | |  |
| **Total:** | | | | | | | | | 3 | | 0 | | |

*Response options include A: accept completely; B: accept with some reservation; C: accept with major reservation; D: reject with reservation; and E: reject completely.

| **1.5. Management of cN1 disease** | | **Response options* (%)** | | | | | **Statement was accepted only if (A + B)% ≥ 80%** | | | | |  |  |
| --- | --- | --- | --- | --- | --- | --- | --- | --- | --- | --- | --- | --- | --- |
| **#** | **Drafted statements** | **A** | **B** | **C** | **D** | **E** | **A + B (%)** | **Accepted** | | **Voted down** | | |  |
| **1** | Neoadjuvant chemotherapy plus perioperative durvalumab IO + RC with pelvic lymph node dissection (i.e. to the level of the ureteric crossing) should be considered in patients with cN1 disease. | 58.33 | 33.33 | 8.33 | 0 | 0 | 91.67 | ✓ | |  | | |  |
| **2** | Pelvic radiation can be considered in patients with cN1 disease who have no progression after systemic therapy. | 33.33 | 58.33 | 8.33 | 0 | 0 | 91.67 | ✓ | |  | | |  |
| **3** | For inoperable locally advanced UC, novel treatments, e.g. enfortumab vedotin + pembrolizumab (EV+P), may be considered. | 91.67 | 8.33 | 0 | 0 | 0 | 100 | ✓ | |  | | |  |
| **Total:** | | | | | | | | | 3 | | 0 | | |

*Response options include A: accept completely; B: accept with some reservation; C: accept with major reservation; D: reject with reservation; and E: reject completely.

| **1.6. Follow-up and monitoring** | | **Response options* (%)** | | | | | **Statement was accepted only if (A + B)% ≥ 80%** | | | | |  |  |
| --- | --- | --- | --- | --- | --- | --- | --- | --- | --- | --- | --- | --- | --- |
| **#** | **Drafted statements** | **A** | **B** | **C** | **D** | **E** | **A + B (%)** | **Accepted** | | **Voted down** | | |  |
| **1** | After RC, patients should be followed up using CT of the thorax, abdomen, and pelvis (TAP) every 3–6 months for 2 years, then every 6–12 months for 3 years, and yearly thereafter. | 58.33 | 33.33 | 8.33 | 0 | 0 | 91.67 | ✓ | |  | | |  |
| **2** | After TMT, patients should be followed up using CT TAP, cystoscopy, and urine cytology every 3–6 months for 3 years and then every 6 months thereafter. | 58.33 | 33.33 | 8.33 | 0 | 0 | 91.67 | ✓ | |  | | |  |
| **Total:** | | | | | | | | | 2 | | 0 | | |

*Response options include A: accept completely; B: accept with some reservation; C: accept with major reservation; D: reject with reservation; and E: reject completely.

**Part 2 – Management of locally advanced UTUC**

| **2.1. Considerations for prescribing neoadjuvant or adjuvant platinum-based chemotherapy or IO in patients with UTUC** | | **Response options* (%)** | | | | | | **Statement was accepted only if (A + B)% ≥ 80%** | | | | | | |
| --- | --- | --- | --- | --- | --- | --- | --- | --- | --- | --- | --- | --- | --- | --- |
| **#** | **Drafted statements** | **A** | **B** | **C** | **D** | **E** | **A + B (%)** | | **Accepted** | | **Voted down** | | | |
| **1** | Adjuvant platinum-based chemotherapy after radical nephroureterectomy (RNU) should be offered to eligible patients with pT2–T4 and/or pN+ disease. | 83.33 | 16.67 | 0 | 0 | 0 | 100 | | ✓ | | |  | | |
| **2** | Carboplatin-based regimens are not suggested in patients who are cisplatin-eligible. | 75 | 16.67 | 8.33 | 0 | 0 | 91.67 | | ✓ | | |  | | |
| **3** | With split doses and hydration, cisplatin may be considered in patients with an estimated GFR down to 45 mL/min/1.73 m^2^. | 58.33 | 33.33 | 8.33 | 0 | 0 | 91.67 | | ✓ | | |  | | |
| **4** | There is currently no high-level evidence to support the use of neoadjuvant IO. | 75 | 25 | 0 | 0 | 0 | 100 | | ✓ | | |  | | |
| **5** | Adjuvant nivolumab therapy can be considered in patients with ypT2–4a or ypN+ UTUC after neoadjuvant cisplatin-based chemotherapy (without IO). | 33.33 | 58.33 | 8.33 | 0 | 0 | 91.67 | | ✓ | | |  | | |
| **6** | Adjuvant pembrolizumab therapy can be considered in patients with ypT2–4a or ypN+ UTUC after neoadjuvant cisplatin-based chemotherapy (without IO). | 16.67 | 58.33 | 25 | 0 | 0 | 75 | |  | | | X | | |
| **7** | Adjuvant nivolumab therapy can be considered in patients with pT3–4a or pN+ UTUC who have not received neoadjuvant cisplatin-based chemotherapy. | 33.33 | 66.67 | 0 | 0 | 0 | 100 | | ✓ | | |  | | |
| **8** | Adjuvant pembrolizumab therapy can be considered in patients with pT3–4a or pN+ UTUC who have not received neoadjuvant cisplatin-based chemotherapy. | 25 | 58.33 | 16.67 | 0 | 0 | 83.33 | | ✓ | | |  | | |
| **9** | There is currently no high-level evidence to support the use of perioperative RT. | 83.33 | 16.67 | 0 | 0 | 0 | 100 | | ✓ | | |  | | |
| **10** | Nuclear and cross-sectional imaging should be performed to help predict the risk and the degree of renal function deterioration after radical surgery. | 58.33 | 41.67 | 0 | 0 | 0 | 100 | | ✓ | | |  | | |
| **11** | Nephron-sparing approaches may be considered in patients with marginal renal function. | 66.67 | 25 | 8.33 | 0 | 0 | 91.67 | | ✓ | | |  | | |
| **12** | In selected patients with marginal renal function, prediction of tumour aggressiveness based on imaging and histology should be performed so that neoadjuvant chemotherapy can be considered for patients with a high-risk tumour. | 58.33 | 33.33 | 0 | 8.33 | 0 | 91.67 | | ✓ | | |  | | |
| **Total:** | | | | | | | | | | 11 | | | 1 |  |

*Response options include A: accept completely; B: accept with some reservation; C: accept with major reservation; D: reject with reservation; and E: reject completely.

| **2.2. Optimal follow-up schedule in patients with UTUC** | | **Response options* (%)** | | | | | | **Statement was accepted only if (A + B)% ≥ 80%** | | | | | | |
| --- | --- | --- | --- | --- | --- | --- | --- | --- | --- | --- | --- | --- | --- | --- |
| **#** | **Drafted statements** | **A** | **B** | **C** | **D** | **E** | **A + B (%)** | | **Accepted** | | **Voted down** | | | |
| **1** | For high-risk tumours post-RNU, cross-sectional imaging of the abdomen and pelvis, preferably with CT urography, should be performed every 6–12 months for years 1–2, and then annually for years 3–4. | 58.33 | 41.67 | 0 | 0 | 0 | 100 | | ✓ | | |  | | |
| **2** | Chest imaging, preferably with CT of the thorax, should be performed every 6–12 months for the first 3–4 years. | 66.67 | 33.33 | 0 | 0 | 0 | 100 | | ✓ | | |  | | |
| **3a** | The following can be considered as a risk factor that may prompt more stringent follow-up schedules:  History of nephron-sparing surgery | 91.67 | 8.33 | 0 | 0 | 0 | 100 | | ✓ | | |  | | |
| **3b** | The following can be considered as a risk factor that may prompt more stringent follow-up schedules: Smoking | 50 | 41.67 | 8.33 | 0 | 0 | 91.67 | | ✓ | | |  | | |
| **3c** | The following can be considered as a risk factor that may prompt more stringent follow-up schedules: Metabolic syndrome | 25 | 50 | 16.67 | 8.33 | 0 | 75 | |  | | | X | | |
| **3d** | The following can be considered as a risk factor that may prompt more stringent follow-up schedules: Obesity | 8.33 | 66.67 | 16.67 | 8.33 | 0 | 75 | |  | | | X | | |
| **3e** | The following can be considered as a risk factor that may prompt more stringent follow-up schedules: Non-UC histology variant | 75 | 25 | 0 | 0 | 0 | 100 | | ✓ | | |  | | |
| **Total:** | | | | | | | | | | 5 | | | 2 |  |

*Response options include A: accept completely; B: accept with some reservation; C: accept with major reservation; D: reject with reservation; and E: reject completely.

**Part 3 – Management of unresectable locally advanced or metastatic UC (mUC)**

| **3.1. Initial treatment choice** | | **Response options* (%)** | | | | | | **Statement was accepted only if (A + B)% ≥ 80%** | | | | | | |
| --- | --- | --- | --- | --- | --- | --- | --- | --- | --- | --- | --- | --- | --- | --- |
| **#** | **Drafted statements** | **A** | **B** | **C** | **D** | **E** | **A + B (%)** | | **Accepted** | | **Voted down** | | | |
| **1** | EV+P is the preferred treatment regimen over platinum-based chemotherapy in eligible patients. | 91.67 | 8.33 | 0 | 0 | 0 | 100 | | ✓ | | |  | | |
| **2a** | Preferred treatment options for cisplatin-eligible patients if EV+P is unavailable or contraindicated include: Gemcitabine-cisplatin chemotherapy and, if there is no disease progression, followed by avelumab maintenance | 83.33 | 16.67 | 0 | 0 | 0 | 100 | | ✓ | | |  | | |
| **2b** | Preferred treatment options for cisplatin-eligible patients if EV+P is unavailable or contraindicated include: Gemcitabine-cisplatin chemotherapy + nivolumab | 66.67 | 33.33 | 0 | 0 | 0 | 100 | | ✓ | | |  | | |
| **3** | The preferred treatment option for cisplatin-ineligible patients is gemcitabine-carboplatin chemotherapy and, if there is no disease progression, followed by avelumab maintenance if EV+P is unavailable or contraindicated. | 100 | 0 | 0 | 0 | 0 | 100 | | ✓ | | |  | | |
| **4** | In patients who received prior adjuvant IO, the clinical benefits of IO-containing regimens as first-line treatment for mUC remain undetermined. | 83.33 | 16.67 | 0 | 0 | 0 | 100 | | ✓ | | |  | | |
| **5** | If EV+P is unavailable or contraindicated, pembrolizumab can be considered for platinum-ineligible patients with PD-L1–positive tumours. | 66.67 | 33.33 | 0 | 0 | 0 | 100 | | ✓ | | |  | | |
| **Total:** | | | | | | | | | | 6 | | | 0 |  |

*Response options include A: accept completely; B: accept with some reservation; C: accept with major reservation; D: reject with reservation; and E: reject completely.

| **3.2. Subsequent treatment approach** | | **Response options* (%)** | | | | | | **Statement was accepted only if (A + B)% ≥ 80%** | | | | | | |
| --- | --- | --- | --- | --- | --- | --- | --- | --- | --- | --- | --- | --- | --- | --- |
| **#** | **Drafted statements** | **A** | **B** | **C** | **D** | **E** | **A + B (%)** | | **Accepted** | | **Voted down** | | | |
| **1** | For patients who progress on EV+P, standard platinum-based chemotherapy without maintenance IO should be considered. | 66.67 | 33.33 | 0 | 0 | 0 | 100 | | ✓ | | |  | | |
| **2** | For patients who progress on EV+P and have *FGFR*-altered tumours, erdafitinib may be considered. | 83.33 | 16.67 | 0 | 0 | 0 | 100 | | ✓ | | |  | | |
| **3** | For patients who progress on platinum-based chemotherapy and avelumab maintenance, subsequent-line treatment options include EV, erdafitinib (for *FGFR*-altered tumours), or chemotherapy. | 83.33 | 16.67 | 0 | 0 | 0 | 100 | | ✓ | | |  | | |
| **4** | In patients who had prior durable responses to platinum-based regimens, rechallenge with platinum-based chemotherapy may be considered. | 50 | 33.33 | 16.67 | 0 | 0 | 83.33 | | ✓ | | |  | | |
| **5** | For patients who progress after IO, rechallenge with a PD-1 or PD-L1 inhibitor is not recommended. | 66.67 | 33.33 | 0 | 0 | 0 | 100 | | ✓ | | |  | | |
| **6** | The choice of subsequent therapy should be individualised based on patient performance status, prior treatment responses and tolerability, and biomarker status. | 100 | 0 | 0 | 0 | 0 | 100 | | ✓ | | |  | | |
| **Total:** | | | | | | | | | | 6 | | | 0 |  |

*Response options include A: accept completely; B: accept with some reservation; C: accept with major reservation; D: reject with reservation; and E: reject completely.

| **3.3. Management of oligometastatic bladder cancer (OMBC)** | | **Response options* (%)** | | | | | | **Statement was accepted only if (A + B)% ≥ 80%** | | | | | | |
| --- | --- | --- | --- | --- | --- | --- | --- | --- | --- | --- | --- | --- | --- | --- |
| **#** | **Drafted statements** | **A** | **B** | **C** | **D** | **E** | **A + B (%)** | | **Accepted** | | **Voted down** | | | |
| **1** | OMBC is defined as having ≤ 3 metastatic sites that are resectable or amenable to stereotactic therapy. | 33.33 | 66.67 | 0 | 0 | 0 | 100 | | ✓ | | |  | | |
| **2** | Systemic therapy remains the mainstay of treatment for OMBC. | 83.33 | 16.67 | 0 | 0 | 0 | 100 | | ✓ | | |  | | |
| **3** | Stereotactic body RT to the metastatic site may be considered in patients with OMBC. | 66.67 | 25 | 8.33 | 0 | 0 | 91.67 | | ✓ | | |  | | |
| **4** | Metastasectomy may be considered in highly selected patients with OMBC. | 50 | 41.67 | 0 | 8.33 | 0 | 91.67 | | ✓ | | |  | | |
| **Total:** | | | | | | | | | | 4 | | | 0 |  |

*Response options include A: accept completely; B: accept with some reservation; C: accept with major reservation; D: reject with reservation; and E: reject completely.
